# TREM2 plays a critical role in innate immune defense against acute *Toxoplasma gondii* infection by promoting macrophage antimicrobial activities

**DOI:** 10.1371/journal.ppat.1014462

**Published:** 2026-08-05

**Authors:** Hannah Z. Debray, Dillon Cheng, L. Angel Ayala, Toan Lam, Ji-Hun Shin, Dequina A. Nicholas, Matthew A. Inlay, Nir Drayman, Robert A. Edwards, Melissa B. Lodoen

**Affiliations:** 1 Department of Molecular Biology and Biochemistry, University of California, Irvine, Irvine, California, United States of America; 2 Institute for Immunology, University of California, Irvine, California, United States of America; 3 Sue and Bill Gross Stem Cell Research Center, University of California, Irvine, California, United States of America; 4 Department of Pathology, University of California, Irvine, School of Medicine, Irvine, California, United States of America; University of California Riverside, UNITED STATES OF AMERICA

## Abstract

The TREM2 receptor is a well-known rheostat for inflammation and immunity, but its role in host defense against parasitic infection is only just emerging. We investigated the function of TREM2 during acute *Toxoplasma gondii* infection by comparing TREM2-deficient and C57BL/6 wild-type (WT) mice during intraperitoneal infection with type II (*Prugniaud* strain) *T. gondii*. Infected TREM2 knockout (KO) mice had significantly increased mortality and elevated parasite burden during acute infection, as well as increased liver pathology and higher levels of inflammatory cytokines IL-1α, IL-6, and IL-17A by 7 days post-infection (dpi). Notably, we observed an early expansion and dissemination of *T. gondii* in infected macrophages in the omentum in TREM2 KO compared to WT mice, and this phenotype was specific to TREM2 deficiency on radiation-sensitive cells, based on bone marrow chimera experiments. *In vitro*, TREM2 KO macrophages were more permissive to *T. gondii* infection and exhibited reduced LAMP1 upregulation and impaired phagocytotic clearance of *T. gondii* compared to WT macrophages. TREM2 deficiency has been previously associated with elevated ERK signaling and defective lysosomal activity, and we found that treatment of TREM2 KO macrophages with the ERK inhibitor SCH772984 rescued LAMP1 expression and improved macrophage control of the parasites. Finally, RNA sequencing of myeloid cells isolated from the peritoneal cavity of infected mice at 3 dpi revealed increased transcripts associated with inflammation and decreased transcripts associated with cellular migration in the TREM2 KO compared to WT mice. These findings demonstrate a critical role for TREM2 in the early antimicrobial immune response to *T. gondii* infection by limiting parasite expansion, dissemination, and pathological inflammation in the infected host.

## Author summary

An effective immune response against infection requires pathogen sensing and control to mitigate damage from overwhelming pathogen burden and inflammation. TREM2 is an immunoreceptor that is highly expressed on myeloid cells, including macrophages, and plays a key role in immune responses during infection and injury. We investigated the role of TREM2 in host defense against the intracellular parasite *Toxoplasma gondii.* Mice deficient in TREM2 were acutely susceptible to *T. gondii* infection, with increased mortality and weight loss compared to wild-type mice. Macrophages from TREM2 knockout mice (KO) had impaired antimicrobial effector functions, and the TREM2 KO mice failed to contain the infection*,* resulting in a rapid dissemination beyond the site of infection and an increase in *T. gondii* parasite burden within macrophages. These deficits in immune control were associated with elevated inflammation and pathology and highlight a key role for TREM2 in early immune defense against *T. gondii* infection.

## Introduction

*Toxoplasma gondii* is one of the most common parasitic infections in humans, with an estimated seropositivity of 22.5% in the U.S., 30–50% in Middle Eastern and European countries, and 80% in South American countries such as Brazil [1–4]. Infection is typically asymptomatic in immunocompetent hosts but can be life-threatening in immunocompromised individuals and the developing fetus during congenital infection [5–7]. Initial control of *T. gondii* infection in the healthy host is highly dependent on the production of IFN-γ [8], which is activated by IL-12 release from dendritic cells [9]. Tissue-resident macrophages, as well as neutrophils and monocytes that are recruited to the site of infection, contribute to parasitic control via phagocytosis, antimicrobial effector mechanisms, and the production of cytokines and chemokines [10–13]. However, the overproduction of inflammatory cytokines, reactive oxygen species (ROS), and nitric oxide (NO) can also cause immunopathology, indicating that antimicrobial responses must be tightly regulated. As a result, a variety of immunoregulatory mechanisms ensure that “first responder” innate cells mount an appropriate inflammatory response to reduce parasite burden, while limiting the potential for immunopathology during the acute stage of infection.

TREM2 (Triggering Receptor Expressed on Myeloid Cells 2) is a cell-surface immunoreceptor that binds to anionic ligands and contributes to a variety of immune functions, including phagocytosis, chemotaxis, and cellular survival [14,15]. TREM2 associates with an ITAM-containing adaptor protein, DAP12, which upon phosphorylation leads to Syk activation and downstream NF-кB signaling and cellular activity [16–18]. TREM2 can also associate with DAP10, which activates PI3K and Akt signaling [19]. In addition, TREM2 can be proteolytically cleaved or alternatively spliced into a soluble form, which has been used as a biomarker of neurodegenerative diseases when detected in the cerebrospinal fluid [20,21]. Although the function of this soluble form of TREM2 is still being elucidated, it is thought to occur after ligand binding to modulate TREM2 signaling [22].

TREM2 is involved in the immune response during various disease conditions and infections. Emerging evidence suggests that TREM2 plays a nuanced role in immunity, as signaling through this receptor can be either detrimental or beneficial to the host, depending on the context. During *Streptococcus pneumoniae* infection, TREM2 was found to inhibit the protective effector functions of complement C1q, such that TREM2 knockout (KO) mice exhibited increased bacterial phagocytosis and clearance in the lung and improved survival compared to their wild-type (WT) counterparts [23]. Similarly, TREM2 KO mice were also protected during *Burkholderia pseudomallei* infection, with reduced bacterial burden, inflammation, and organ injury, and increased survival compared to WT mice [24]. In contrast, TREM2 can also function in a host protective manner by polarizing CD4^+^ T cells into Th1 cells to enhance immune responses to SARS-CoV-2 and *Mycobacterium tuberculosis* infections [25,26]. As a result, TREM2 can play both protective and deleterious roles in immunity, depending on the nature of the pathogen, the site of infection/injury, and the immune environment created by both tissue-resident and recruited immune cells.

A recent paper demonstrated that TREM2 contributes to host protection against *T. gondii*-induced adverse pregnancy outcomes by modulating decidual macrophage responses [27]. However, the effects of TREM2 deficiency on the peripheral immune response against *T. gondii* in non-pregnant adult mice has not been examined. We observed increased susceptibility of TREM2 KO compared to WT mice during *T. gondii* infection, and our data reveal a critical protective role for TREM2 in key antimicrobial innate immune responses during acute *T. gondii* infection.

## Results

### TREM2-deficient mice are more susceptible to *Toxoplasma gondii* infection

**Fig 1:**
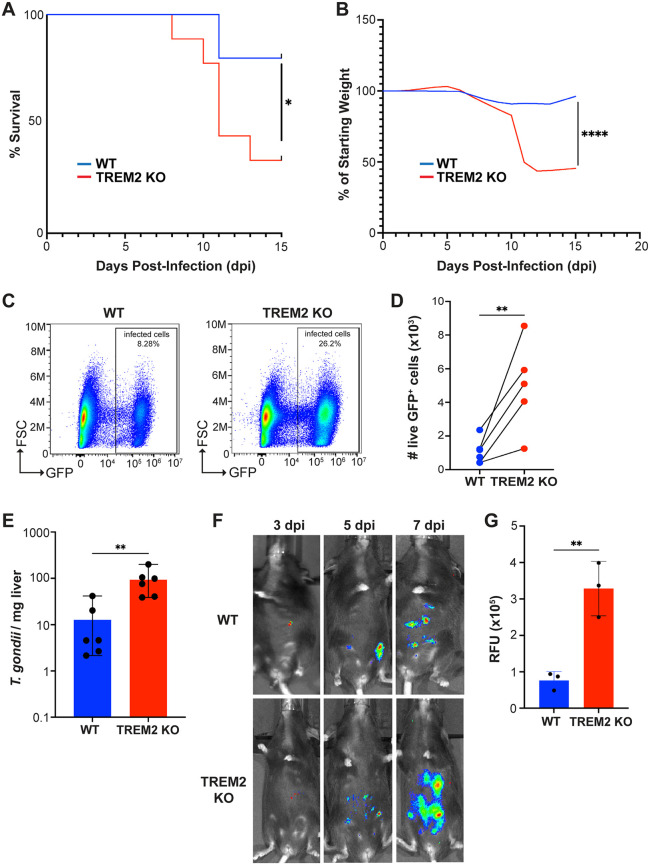
TREM2-deficient mice are more susceptible to *Toxoplasma gondii* infection. C57BL/6 (WT) and TREM2 KO mice were infected intraperitoneally (i.p.) with 200 GFP-expressing type II *Prugniaud* strain *T. gondii* or injected with PBS. **A)** Survival and **B)** Percent weight loss were monitored over time. **C)** Representative flow cytometry plots showing the percent of GFP^+^ (infected cells) from the peritoneal exudate cells (PECs) of WT and TREM2 KO mice at 7 dpi. **D)** Number of live GFP^+^ cells from the PECs of infected WT and TREM2 KO mice. Each point is the average of all of the mice from a given experiment for each genotype (the data reflect 5 independent experiments, and the lines link the WT and TREM2 KO mice from the same experiment). **E)** Assessment of liver parasite burden by qPCR for B1 from WT and TREM2 KO mice at 7 dpi. **F)** Bioluminescent imaging (BLI) of WT and TREM2 KO mice infected with FLUC-expressing *T. gondii* at 3, 5, and 7 dpi. **G)** Quantification of parasite burden in BLI experiments by maximal RFU. In **(A)** n = 15-16 mice per group from 6 independent experiments, compared using Log-rank (Mantel Cox) test. In **(B)** n = 6 mice per group from 2 independent experiments, compared using linear regression. In **(D)** n = 17 mice per group from 5 experiments, compared using randomized block ANOVA. In **(E)** n = 6 mice per group, compared using unpaired Student’s t-test. In **(G)** n = 3 mice per group, compared using Student’s unpaired t-test. ****p < 0.0001, **p < 0.01, *p < 0.05.

To investigate the degree to which TREM2 contributes to the immune response against *T. gondii* infection, we injected C57BL/6 (WT) and TREM2 knockout (KO) mice with 200 green fluorescent protein (GFP)- and firefly luciferase (FLUC)-expressing type II (*Prugniaud* strain) tachyzoites intraperitoneally (i.p.) and analyzed the mice during infection. Although 80% of the WT mice survived acute infection, the TREM2 KO mice began to succumb as early as 8 days post-infection (dpi). Only 40% of the TREM2 KO mice survived to 15 dpi (Fig 1A), and there was significantly lower body weight in the surviving mice (Fig 1B). Since the parasites constitutively expressed GFP, we analyzed the percent of GFP^+^ (infected) cells in the peritoneal cavity of the infected mice at 7 dpi and observed an increased parasite burden in the TREM2 KO mice (Figs 1C and 1D), as well as elevated parasite DNA in the liver (Fig 1E) by 7 dpi. We also detected increased parasite bioluminescent signal in the TREM2 KO mice using an *in vivo* imaging system (IVIS) to detect the FLUC-expressing parasites (Fig 1F and 1G). Collectively, these data indicate that TREM2 KO mice were more susceptible to *T. gondii* infection than WT mice, with increased mortality and parasite burden.

### TREM2 deficiency results in increased inflammation and liver pathology during *T. gondii* infection

**Fig 2:**
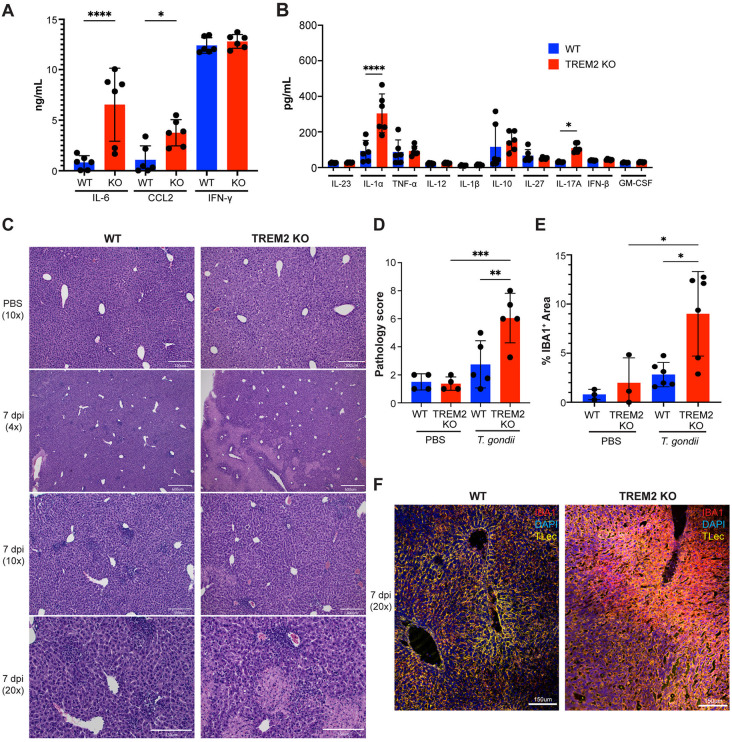
TREM2-deficient mice have increased pathology during *T. gondii* infection. C57BL/6 (WT) and TREM2 KO mice were injected i.p. with PBS as a control or type II *T. gondii* and analyzed at 7 dpi. **A, B)** Cytokine and chemokine levels in peritoneal exudate collected from WT and TREM2 KO mice, with higher concentration analytes graphed in **(A)** and lower concentration analytes in **(B)**. **C)** Hematoxylin and eosin **(H&E)**-stained liver sections from PBS-injected and *T. gondii-*infected mice, imaged at 4x, 10x, and 20x magnifications. **D)** Pathology scores of disease severity in control and infected livers based on H&E staining. **E)** Quantification of % IBA1^+^ area/FOV from confocal microscopy of liver sections from *T. gondii-*infected WT and TREM2 KO mice. **F)** Representative 20x tilescans of confocal images of livers from *T. gondii-*infected WT and TREM2 KO mice. Sections were stained with DAPI (blue), anti-IBA1 (red), and tomato lectin (TLec, yellow). In (**A-B**) n_*T. gondii*_ = 6 mice/genotype from 2 independent experiments. In **(D)** n_PBS_ = 4 mice and n_*T. gondii*_ = 5 mice per genotype from 2 independent experiments. In (**E**) n_PBS_ = 3 mice and n_*T. gondii*_ = 6 mice per genotype from 2 independent experiments, with 3-7 FOVs averaged per mouse. Statistical significance was determined using one-way ANOVA with a post-hoc Tukey test. ****p < 0.0001, ***p < 0.001, **p < 0.01, *p < 0.05.

Since the TREM2 KO mice failed to control acute *T. gondii* infection, we examined whether the cytokine response differed in WT and TREM2 KO mice after i.p. infection. Peritoneal fluid was harvested at 3 and 7 dpi from PBS-injected or *T. gondii*-infected WT and TREM2 KO mice and analyzed by a flow cytometry-based multiplex assay. PBS-injected mice showed very low or non-detectable levels of the 13 analytes (S1A Fig), whereas infection resulted in increased levels of several chemokines and cytokines at 3 and 7 dpi in both genotypes of mice (Figs 2A, 2B and S1B). Notably, there were comparable levels of IFN-γ, a key driver of immune control against *T. gondii* [8], in the WT and TREM2 KO mice at both timepoints (Figs 2A and S1B). In contrast, levels of inflammatory IL-6, CCL2, IL-1α, and IL-17A were significantly higher in the TREM2 KO mice than the WT mice by 7 dpi (Figs 2A and 2B), correlating with the elevated parasite burden in the TREM2 KO mice at this timepoint.

The liver is an early organ of *T. gondii* replication and associated hepatic pathology [28–30], and higher parasite loads were detected in the livers of TREM2 KO mice at 7 dpi (Fig 1E). To investigate the effects of parasite infection and inflammation in this organ, we stained liver tissue sections with hematoxylin and eosin (H&E). Imaging at low and high magnification revealed regions of hepatocyte loss and necrosis in the TREM2 KO mice during infection (Fig 2C). The liver sections were scored by a pathologist in a blinded fashion for disease severity, according to metrics including foci of infiltrating inflammatory cells and hepatocyte damage. The livers from TREM2 KO mice were consistently ranked higher in disease severity, with numerous inflammatory foci and areas of hepatocyte damage and death, indicative of liver infarction (Fig 2C and 2D), whereas the livers from PBS-injected mice showed no pathological difference between the genotypes (Figs 2C and 2D). Liver sections were also examined by immunofluorescence microscopy for IBA1^+^ myeloid cells and for vascular architecture by staining with tomato lectin (TLec). IBA1 is a marker for tissue-resident macrophages in the liver (Kupffer cells) and infiltrating monocytes. We observed significantly increased IBA1 signal and IBA1^+^ cell accumulation in the TREM2 KO compared to WT livers at 7 dpi (Figs 2E and 2F). These findings support a protective role for TREM2 in preventing liver inflammation and pathology during *T. gondii* infection.

### Rapid expansion and dissemination of *T. gondii* occurs in TREM2 KO mice

**Fig 3:**
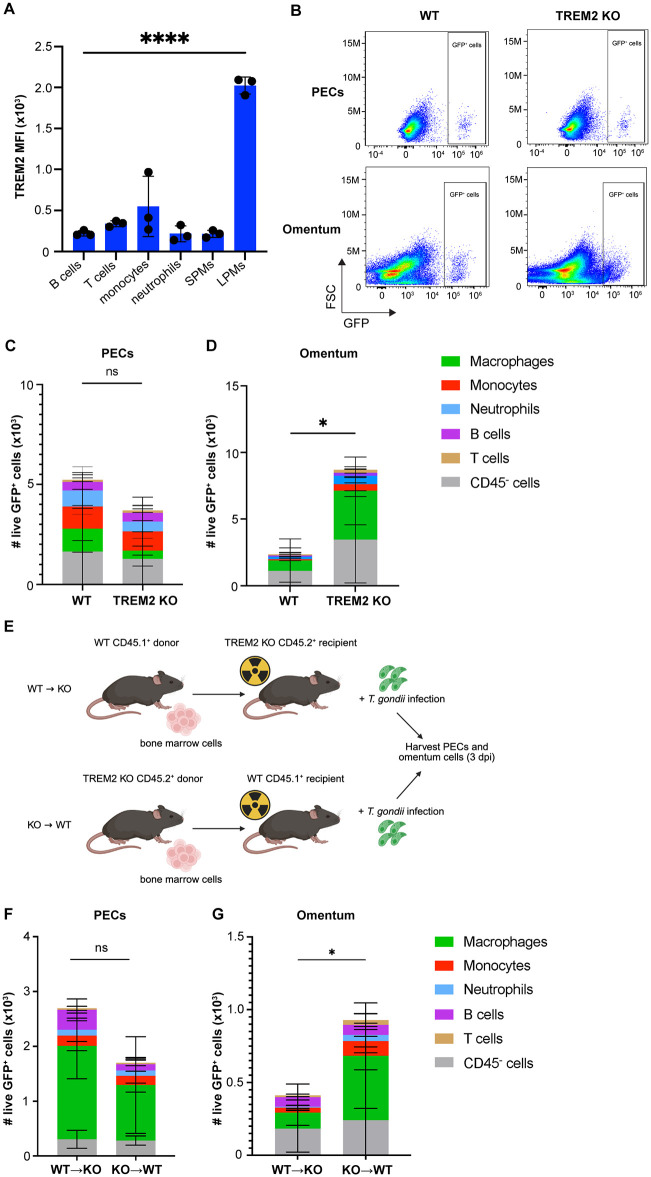
Rapid *T. gondii* dissemination and expansion in parasite numbers occurs in TREM2 KO mice. **A)** Mean fluorescence intensity of TREM2 expression on peritoneal immune cells in PBS-injected WT mice. Neutrophils (CD45^+^ CD11b^+^ Ly6G^+^), monocytes (CD45^+^ CD11b^+^ Ly6C^+^), T cells (CD45^+^ CD3^+^), B cells (CD45^+^ CD19^+^), small peritoneal macrophages or SPMs (CD45^+^ CD11b^+^ F4/80^low^) and large peritoneal macrophages or LPMs (CD45^+^ CD11b^+^ F4/80^high^) were analyzed by flow cytometry. **B)** C57BL/6 (WT) and TREM2 KO mice were infected i.p. with 200 *T. gondii* tachyzoites*.* Representative flow plots of the number of infected (GFP^+^) cells in the PECs and the omentum at 3 dpi. **C-D)** Number of infected (GFP^+^) cells within each immune cell population, in the PECs (**C**) and the omentum (**D**) at 3 dpi. **E)** Schematic of the bone marrow chimera experiment. Recipient mice were irradiated and reconstituted with 5x10^6^ donor bone marrow cells. Following a 10-week engraftment, mice were infected as described above, and PECs and omentum were harvested and analyzed. **F-G)** Number of infected (GFP^+^) cells within each immune cell population, in the PECs (**F**) and the omentum (**G**) at 3 dpi within chimeric mice. Created in BioRender (2026) https://BioRender.com/uqkzs6l. In (**A**) n = 3 mice. In (**C-D**) n_*T. gondii*_ = 12 mice per genotype from 4 independent experiments. In (**F-G**) n_*T. gondii*_ = 4–6 mice per genotype from 2 independent experiments. Statistical significance was determined using one-way ANOVA with a post-hoc Tukey test (**A**), or Student’s t-test (**C-D**, **F-G**). ****p < 0.0001, *p < 0.05.

Given the profound defect in immune control of *T. gondii* by 7 dpi in the TREM2 KO mice, we investigated the immune response at an earlier timepoint to determine the mechanisms underlying the impaired host defense in these mice. Notably, in the peritoneal cavity of WT mice, large peritoneal macrophages (LMPs) expressed the highest levels of TREM2 (Fig 3A). In examining the interactions of *T. gondii* with cells in the peritoneal cavity at 3 dpi, we detected similar numbers of cells containing GFP^+^ parasites in WT and TREM2 KO mice (Figs 3B and 3C), and these infected cells were predominantly myeloid cells, including monocytes, macrophages, and neutrophils in both genotypes of mice (S2A and S2B Figs). The omentum is a specialized adipose structure within the peritoneal cavity comprised of fat-associated lymphoid clusters that function as secondary lymphoid tissues draining the peritoneal cavity [31]. Since large peritoneal macrophages (LPMs) are known to traffic to the omentum after i.p. infection with *T. gondii* [32], we also examined this lymphoid tissue during infection (S3 Fig). Surprisingly, we observed significantly more *T. gondii-*infected cells in the omentum in TREM2 KO mice than in WT mice at 3 dpi (Fig 3D). Among the CD45^+^ infected cells in the omentum, the majority were macrophages, though infected monocytes and neutrophils were also detected (Figs 3D and S3B). These data suggest that the omentum serves as a site of rapid parasite dissemination in the context of TREM2 deficiency, as there was a considerable increase in the number parasite-infected macrophages in the omentum in TREM2 KO mice.

To test whether TREM2 expression on hematopoietic cells was necessary for immune control of *T. gondii* infection *in vivo*, we conducted bone marrow chimera experiments in which WT or TREM2 KO bone marrow was transplanted into lethally irradiated WT or TREM2 KO recipient mice prior to infection (Fig 3E). We confirmed that > 95% of the peripheral blood cells in the recipient mice were donor-derived, indicating successful engraftment (S4A and S4B Fig). In addition, the LPMs in the peritoneal cavity of naïve transplanted mice were also derived from donor bone marrow (S4C and S4D Fig). At 3 dpi, there was not a statistically significant difference in the number of GFP^+^ *T. gondii-*infected cells in the PECs between either set of chimeric mice (Fig 3F). However, WT mice reconstituted with TREM2 KO bone marrow had increased numbers of *T. gondii-*infected cells in the omentum compared to WT mice that received WT bone marrow (S4F Fig) or to TREM2 KO mice that received WT bone marrow (Fig 3G). Thus, WT mice with TREM2 deficiency in the transplanted cells recapitulated the phenotype of high parasite burden in the omentum, similar to the whole body TREM2 KO mice. These findings support the hypothesis that TREM2 expression on radiation-sensitive cells is critical for controlling parasite burden during acute *T. gondii* infection.

### TREM2-deficient macrophages have impaired antimicrobial responses to infection

**Fig 4:**
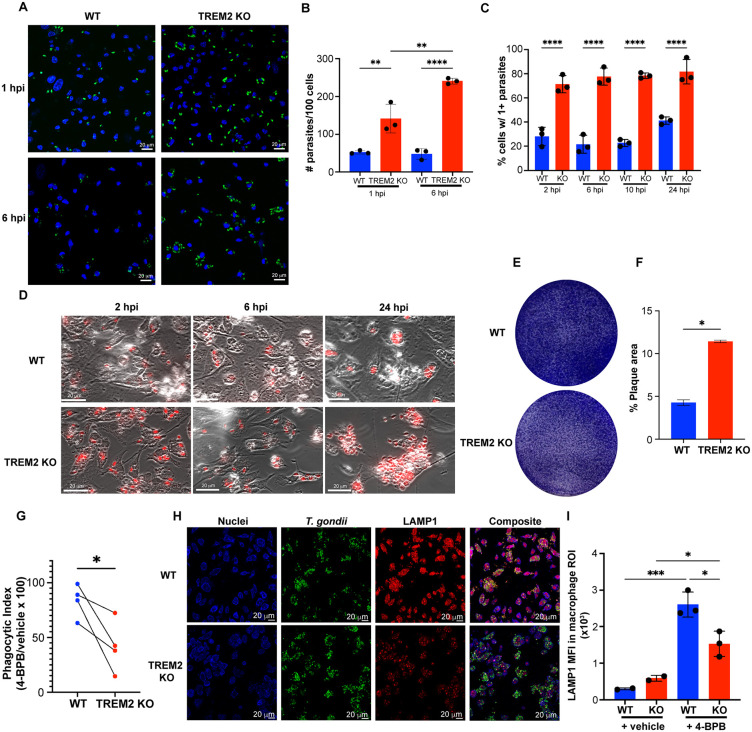
TREM2-deficient macrophages have increased parasite burden and decreased LAMP1 expression. Bone marrow-derived macrophages (BMDMs) from naïve C57BL/6 (WT) and TREM2 KO mice were infected with *T. gondii* at a MOI of 2. **A)** Representative 63X confocal images of WT and TREM2 KO BMDM infected with GFP^+^ *T. gondii* (green) for 1 hr or 6 hr and stained with DAPI (blue). **B)** Quantification of *T. gondii* parasites per 100 cells from the experiments in **(A)**. **C)** Percent of BMDM with at least 1 parasite/cell from the experiments in **(D)**. **D)** Representative 63X images of WT and TREM2 KO BMDMs infected over time with tdTomato^+^ *T. gondii* at a MOI of 2. **E)** Image of HFF plaque assay using parasites isolated from *T. gondii-*infected WT and TREM2 KO BMDM at 24 hpi. **F)** Percent of plaque area from the experiments in **(E)**. **G)** Phagocytic index of WT or TREM2 KO BMDM as determined by the ratio of cells containing 4-BPB-treated parasites (phagocytosis) relative to vehicle-treated parasites (invasion). **H)** Confocal microscopy of F4/80 (blue), *T. gondii* (green), and LAMP1 (red) in 40X images of WT or TREM2 KO BMDMs incubated with 4-BPB-treated *T. gondii* (MOI = 10) for 1 hr. **I)** Quantification of LAMP1 mean fluorescence intensity (MFI) within F4/80 signal from WT or TREM2 KO BMDM incubated with vehicle- or 4-BPB-treated *T. gondii* at a MOI of 10. In (**A**) n = 3 experiments/group with 3-5 FOV each. In (**B-D**) n = 3 experiments/group with 5 FOV each. In (**E-F**) n = 2 experiments. In (**G**) n = 4 experiments. In (**I**) n = 2-3 experiments/group, with 3-5 FOV each. Statistical significance was determined using a one-way ANOVA with a post-hoc Tukey test (**B, C, I**), Student’s t-test (**F**), and paired t-test (**G**). ****p < 0.0001, ***p < 0.001, **p < 0.01, *p < 0.05.

Since TREM2 is most highly expressed on LPMs in the peritoneal cavity, we hypothesized that the increased susceptibility of TREM2 KO mice to *T. gondii* infection was due to impaired macrophage antimicrobial responses to the parasite infection. To test this hypothesis more mechanistically, we turned to an *in vitro* model to compare the infection of WT and TREM2 KO bone marrow-derived macrophages (BMDM) with GFP-expressing *T. gondii* by confocal microscopy (Fig 4A). At both 1 hpi and 6 hpi, there were significantly more parasites in TREM2 KO compared to WT macrophages (Fig 4B). A longer time-course analysis revealed that the percent of infected TREM2 KO BMDM was consistently higher than WT BMDM at all time points out to 24 hpi (Fig 4C), with the TREM2 KO BMDM harboring more parasites per cell (Fig 4D). Moreover, plaque assays conducted with *T. gondii* harvested from infected TREM2 KO BMDM resulted in significantly greater plaque area than when parasites were harvested from WT BMDM (Figs 4E and 4F). These data demonstrate that TREM2 KO macrophages are more permissive to *T. gondii* infection and result in more infectious parasites than WT macrophages.

When *T. gondii* interact with phagocytes, the parasites can either invade the cells and establish a productive infection, or they can be phagocytosed and degraded. Since TREM2 has been shown to be involved in macrophage phagocytosis and ROS production [33,34], we hypothesized that the TREM2 KO macrophages were impaired in phagocytosing *T. gondii*. To examine macrophage phagocytic potential, we utilized 4-bromophencyl bromide (4-BPB), a phospholipase inhibitor that prevents *T. gondii* invasion [35] and results in parasite phagocytosis by macrophages [36]*.* In BMDM cultured with vehicle control-treated tdTomato-expressing *T. gondii*, the tdTomato signal was detectable within the cells at 1 hpi and maintained at 24 hpi, indicative of productive infection (S5A and S5B Fig). In contrast, WT BMDM cultured with 4-BPB-treated parasites had detectable tdTomato signal at 1 hr but significantly less tdTomato signal at 24 hr, consistent with the intracellular parasites losing fluorescence due to parasite phagocytosis and degradation (S5A and S5B Fig). As an additional control for the efficacy of the drug treatment, plaque assays using *T. gondii* harvested from 4-BPB-treated parasites resulted in no detectable plaques (S5C Fig). Notably, when we compared the phagocytic index of WT or TREM2 KO BMDM, we observed significantly less phagocytosis of *T. gondii* by the TREM2 KO macrophages (Fig 4G). These results aligned with a defect in TREM2 KO macrophage phagocytosis of pHrodo *E. coli*, which are heat-killed bacteria conjugated to a fluorogenic reagent that fluoresces in the low pH environment of the phagosome (S5D Fig).

To more directly assess a marker of phagolysosomal activation, we cultured WT and TREM2 KO BMDM with GFP-expressing 4-BPB-treated *T. gondii* and examined the cells by confocal microscopy for LAMP1 signal within F4/80-expressing cells after 1 hr of culture. TREM2 KO macrophages had significantly less LAMP1 signal than WT macrophages cultured with 4-BPB-treated *T. gondii* (Figs 4H and 4I). In contrast, in WT and TREM2 KO BMDM infected with *T. gondii* that were capable of invasion, LAMP1 signal was comparably low (Fig 4I)*.*

**Fig 5:**
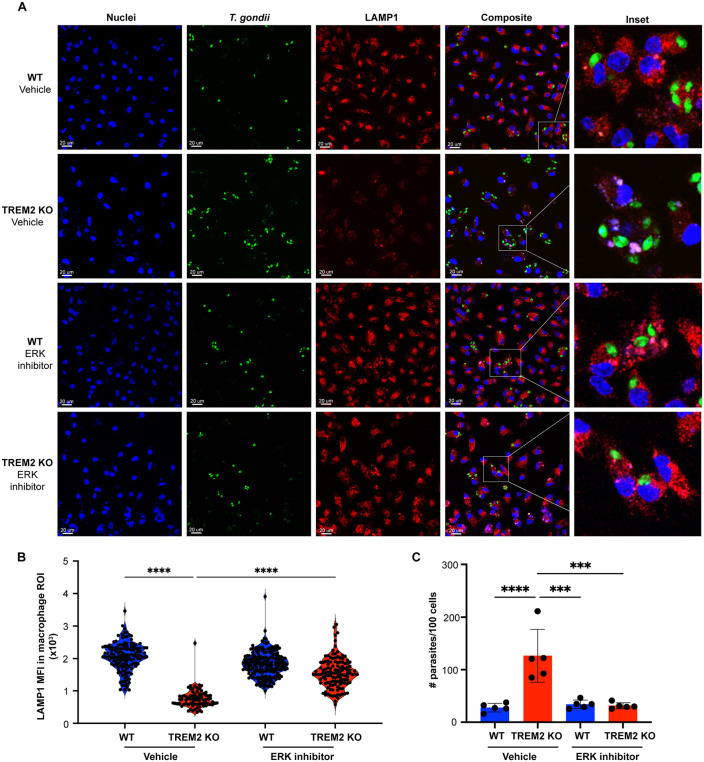
ERK inhibition rescues LAMP1 expression and reduces parasite burden in TREM2 KO macrophages. Bone marrow-derived macrophages (BMDMs) from naïve C57BL/6 (WT) and TREM2 KO mice were pre-treated with ERK inhibitor SCH772984 for 2 hr, washed, and incubated for 6 hr with GFP^+^ *T. gondii* at a MOI of 2. A) Representative 63X confocal images of *T. gondii-*infected WT and TREM2 KO BMDM with DAPI (blue), LAMP1 (red), and *T. gondii* (green). B) Quantification of LAMP1 mean fluorescence intensity (MFI) within F4/80 signal. C) Number of parasites per 100 cells from experiments in (A). In (**A-B**) n = 100-150 cells from 2 independent experiments/group. In **(C)** n = 5 independent experiments. Statistical significance was determined using a one-way ANOVA with a post-hoc Tukey test. ****p < 0.0001, ***p < 0.001.

TREM2 influences lysosomal function and autophagy, including the expression of lysosome-associated genes, via the transcription factor TFEB [37]. Recent work has shown that in TREM2 KO cells, increased ERK1/2 activity dysregulates TFEB, reducing lysosomal gene expression and function in microglia [37]. To examine whether a similar pathway may account for the impaired phagolysosomal degradation of *T. gondii* in TREM2 KO macrophages, we treated WT or TREM2 KO BMDM with the ERK inhibitor SCH772984 and examined LAMP1 expression at 6 hpi (Fig 5A). Although ERK inhibition did not affect the expression of LAMP1 in WT cells during *T. gondii* infection, there was a significant increase in LAMP1 expression in TREM2 KO macrophages treated with the ERK inhibitor (Figs 5A and 5B). Notably, ERK inhibition of TREM2 KO macrophages also resulted in reduced parasite burden, which was restored to the levels observed in WT macrophages (Figs 5A and 5C). Similar results were also observed as early as 1 hpi (S6 Fig). Collectively, these data suggest that impaired *T. gondii* control by TREM2-deficient macrophages is due to ERK-mediated regulation of phagolysosomal activity.

### Myeloid cells from TREM2-deficient mice have differential expression of genes involved in inflammation and innate responses

**Fig 6:**
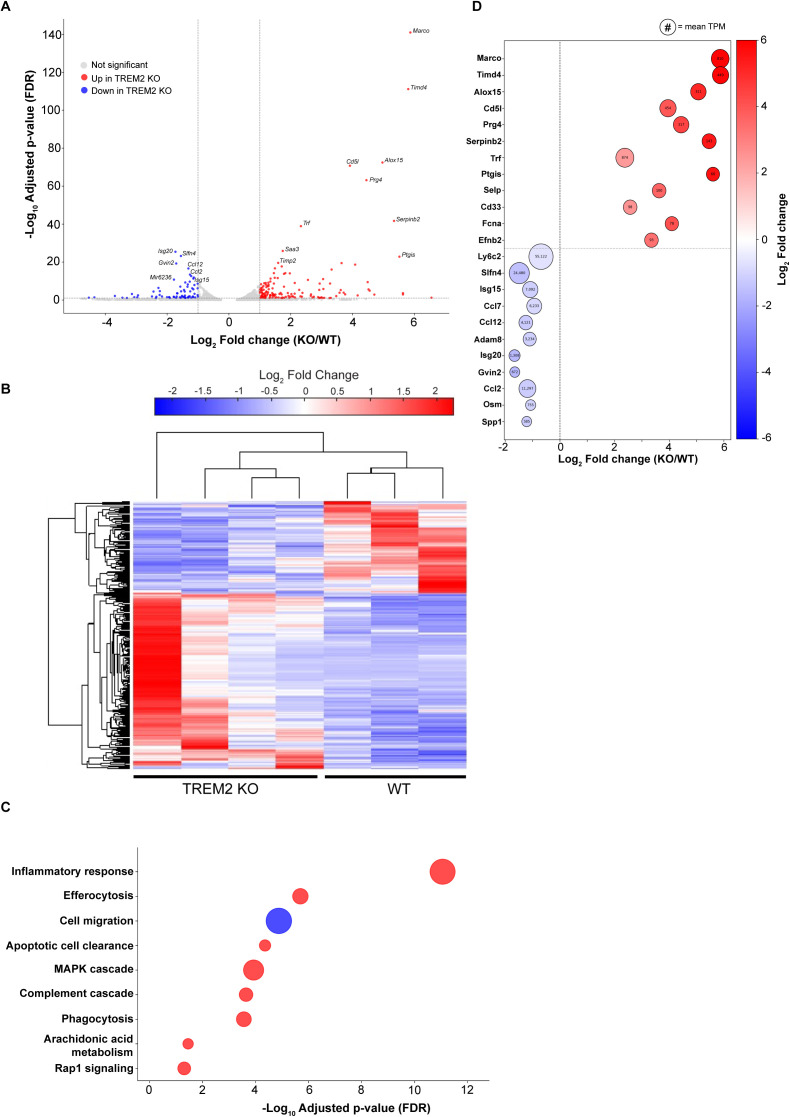
Myeloid cells from TREM2-deficient mice at 3 dpi display differentially expressed genes (DEGs) involved in chemotaxis and inflammation. C57BL/6 (WT) and TREM2 KO mice were infected i.p. with *T. gondii*. The peritoneal exudate cells were harvested at 3 dpi and sorted for CD45^+^CD11b^+^Ly6C^+^ monocytes, and bulk RNAseq was performed. **A)** Volcano plot of transcripts that were upregulated (red dots) or downregulated (blue dots) in monocytes from the infected KO compared to infected WT mice. **B)** Heatmap of all DEGs in monocytes from infected KO relative to infected WT mice based on hierarchical clustering. **C)** Functional enrichment analysis of DEGs showing significantly different pathways. The size of the bubbles reflects the number of genes identified in each pathway, and the colors reflect whether the pathway is up (red) or down (blue) in KO mice. **D)** Differences in expression of the genes most highly expressed in KO relative to WT (red) and the genes most highly expressed in WT relative to KO (blue). DESEQ2 identification of fold change (1.5 cutoff) and significance (< 0.05) filters were used. TPMs are shown within each bubble and reflect the bubble size. n = 3 mice (WT) or 4 mice (TREM2 KO).

To further investigate the early immune response in TREM2 KO mice during *T. gondii* infection in an unbiased manner, we conducted genome-wide transcriptomic profiling of myeloid cell immune cells at the site of infection. WT or TREM2 KO mice were infected i.p. as above, and at 3 dpi, the peritoneal exudate cells were sorted for CD45^+^CD11b^+^Ly6C^+^ monocytes and processed for RNA sequencing (see S7 Fig for experimental workflow, cell sorting plots, and PCA analysis). We identified 272 differentially expressed genes (DEGs) between the WT and TREM2 KO infected monocytes based on an absolute fold-change >1.5 and FDR < 0.05 (Figs 6A and 6B). A gene ontology enrichment analysis revealed that the cell migration pathway was reduced in the TREM2 KO mice, whereas inflammatory response, MAPK signaling, and complement cascade were all increased in the TREM2 KO mice (Fig 6C). We also noted decreased expression of several chemokine genes, including transcripts for *Ccl2, Ccl7*, and *Ccl12* (Fig 6D) in cells from the infected TREM2 KO mice relative to the infected WT mice, suggesting a possible defect in chemotactic recruitment of innate immune cells to the site of infection. Top upregulated genes in monocytes from TREM2 KO mice included genes involved in scavenging, lipid handling, and alternative activation, which may suggest potential compensation by recruited monocytes due to TREM2 deficiency in the resident peritoneal macrophages. Altogether, these data reveal that myeloid cells from TREM2 KO mice expressed higher expression of genes associated with inflammation and efferocytosis, but transcripts for key proteins involved in innate immune cell chemotaxis and interferon stimulation were notably reduced in cells from the TREM2 KO compared to WT mice during infection.

## Discussion

TREM2 is a member of a large family of TREM receptors that modulate the immune response [38]. During infection, TREM2 has been ascribed both protective and detrimental roles for the host, depending on the context of the infection [23–26,39]. A variety of reasons may account for these seemingly opposing functions of TREM2: the expression of TREM2 by both immune and non-immune cells, its ability to bind many different ligands with varying affinity/avidity [19], and the soluble and transmembrane forms of TREM2 that confer different signals from the cell membrane [22]. TREM2 has also been implicated in neurodegenerative diseases, such as Alzheimer’s disease, in which a unique TREM2 variant has been linked to accelerated AD progression and impaired microglial chemotaxis towards amyloid plaques [40]. We found that TREM2 plays a protective role in host defense against *T. gondii* infection, and that TREM2 deficiency was associated with impaired antimicrobial responses and increased parasite burden and inflammation.

Our findings are consistent with a recent study examining the role of TREM2 during *T. gondii* infection of pregnant female mice, which showed that TREM2-deficient mice had more severe pathology, adverse pregnancy outcomes, and higher parasite burden in the placenta [27]. This study by Wang *et. al* also reports that BMDM from TREM2 KO mice release higher levels of inflammatory cytokines but reduced amounts of CXCL1, a neutrophil chemoattractant for microbial killing at the tissue site [27,41]. We have identified the same phenotype *in vivo*, with increased inflammation but significantly lower expression of CC and CXC chemokine family genes in TREM2 KO compared to WT monocytes from infected mice. These independent observations reinforce the emerging view that TREM2 serves as a regulatory node in innate immune function, in which inflammatory cytokine release may be uncoupled from chemokine production.

The TREM2/DAP12 signaling axis has been linked to both Syk and PLCγ activation, which in turn facilitate cell migration and inflammation [42], and we have previously shown that *T. gondii* infection activates Syk signaling [43]. Our transcriptomic analysis on myeloid cells at the site of *T. gondii* infection revealed different responses of TREM2 KO and WT monocytes to infection, with TREM2 KO monocytes adopting a potentially compensatory program, whereas WT monocytes showed a classical inflammatory, interferon-driven activation. GATA6, a master transcription factor selectively expressed in large peritoneal macrophages, was also upregulated in recruited TREM2 KO monocytes (though it was not among the top upregulated genes) [44]. Monocytes are known to replenish the peritoneal macrophage niche throughout adulthood in response to microbial exposure, so this differential gene expression could be attributed to increased parasite burden caused by the impaired TREM2 KO macrophage antimicrobial responses [45]. As large peritoneal macrophages are the first to encounter pathogens in models of intraperitoneal infection, these cells play a critical role in antimicrobial functions and in recruiting other innate immune cells to the site of infection [46].

In our model we infect mice with *T. gondii* tachyzoites in the peritoneal cavity, which enables the injection of a precise dose of tachyzoites, though oral infection with bradyzoite-containing tissue cysts is a more physiological route of *T. gondii* infection. Since TREM2 is expressed on intestinal macrophages [47,48], investigating the outcome of oral *T. gondii* infection of TREM2 KO mice may also reveal a unique function for TREM2 in the gut mucosa. Nonetheless, our current research expands on the work of Wang *et al.* by showing the impact of TREM2 deficiency in the peripheral immune response of male and female adult mice and the functional implications for immune defense at the site of infection and in distal organs during acute infection.

**Fig 7:**
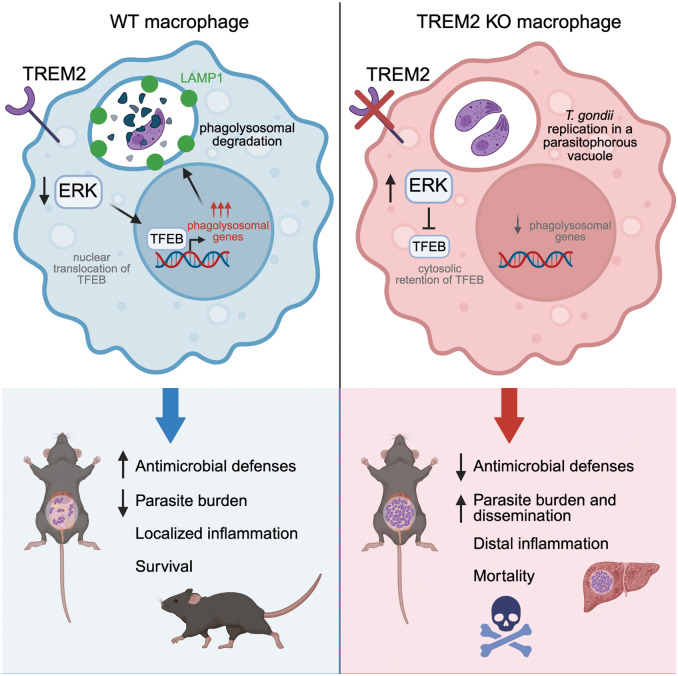
Model for the impact of TREM2 deficiency on macrophage control of *T. gondii* infection and inflammation in the host. TREM2 receptor activation limits intracellular ERK signaling and enables nuclear translocation of TFEB to increase phagolysosomal activity in WT macrophages. During *T. gondii* infection, WT macrophages (left) can form an effective phagolysosome marked by LAMP1 to control the parasites. In contrast, TREM2-deficient macrophages (right) may have increased ERK activity and cytosolic retention of TFEB, which decreases their phagocytic clearance of *T. gondii*. Increased parasite replication in TREM2 KO cells leads to elevated parasite burden and dissemination. TREM2 KO mice also exhibit overwhelming inflammation and increased mortality compared to WT mice during *T. gondii* infection. Created in BioRender (2026) https://BioRender.com/1a7mm16.

We also observed a marked dysregulation of key antimicrobial functions in TREM2 KO macrophages during *T. gondii* infection. In particular, TREM2 KO macrophages exhibited reduced phagocytic clearance of *T. gondii.* These findings align with prior research linking the absence of TREM2 to deficits in phagocytosis and ROS production [33,34]. Notably, ERK inhibition in TREM2 KO macrophages was able to rescue the antimicrobial control of the parasites, potentially via upregulation of phagolysosomal activation. *In vivo*, impaired macrophage effector functions in TREM2 KO mice may lead to reduced immune control of *T. gondii* in the peritoneal cavity. Indeed, we observed rapid *T. gondii* dissemination and an increase in parasite numbers in macrophages in the omentum of *T. gondii-*infected TREM2 KO mice at a very early timepoint (3 dpi). These data support a model whereby large peritoneal macrophages in TREM2 KO mice fail to control/contain the infection, resulting in increased parasite dissemination to the omentum and an early expansion of parasite numbers within macrophages (Fig 7). As a result, incoming monocytes may increase the expression of inflammatory genes in response to the parasite infection or to compensate for the impaired response of resident peritoneal macrophages. TREM2 expression on radiation-sensitive cells, including peritoneal and bone marrow-derived macrophages, was essential for early immune control of *T. gondii* infection. However, the current findings do not rule out the possibility that TREM2 expression or function on other hematopoietic cell types contributes to optimal control of the parasites, and further research using mice with macrophage-specific deletion of TREM2 would more definitively address this possibility.

One question that emerges from our studies is the relative importance of TREM2 in immunoregulation versus innate immune control of the infection. In macrophages, TREM2 was initially described as a negative regulator of TLR and FcR signaling that plays an important role in dampening inflammatory immune responses [16,49]. Indeed, we observed increased inflammatory pathways in TREM2 KO mice during *T. gondii* infection, and TREM2-deficient mice produced significantly more CCL2, IL-6, IL-17A, IFN-β, and IL-1α. TREM2 deficiency also resulted in increased mortality and parasite burden in mice and heightened pathology in the liver. This phenotype is reminiscent of that observed in non-alcoholic fatty liver disease due to inflammation [50]. Increased pathology, along with bleeding and necrosis, were also detected in the placenta of TREM2 KO mice by Wang *et. al.* during *T. gondii* infection of pregnant dams [27]. During innate immune responses to *T. gondii* in the mouse, TLR11/12 recognize the parasite protein profilin, which drives IL-12 production by dendritic cells [51], leading to T cell IFN-γ production. Given the known role of TREM2 as a negative regulator of TLR signaling, the increased inflammation observed in TREM2 KO mice during *T. gondii* infection may be due to reduced inhibition of TLR11/12-mediated immune activation, resulting in impaired immunoregulation in the TREM2 KO mice. The inability of TREM2 KO innate immune cells to mount an effective host response, as discussed above, may also allow *T. gondii* to replicate unchecked, leading to increased inflammation. Transcripts for CD33, which is a sialic acid-binding inhibitor receptor with ITIM domains [52], were increased in cells from TREM2 KO cells by RNAseq analysis. Ligation of CD33 recruits SHP1 phosphatase, which can block Syk signaling downstream of TREM2 activation [53]. Both CD33 and TREM2 functionally interact with DAP12/TYROBP, and share signaling molecules, so loss of one receptor may affect the expression or function of the other [52,54], thereby influencing the balance of immune regulation or inflammation during infection. Collectively, this research contributes to a growing body of evidence indicating that TREM2 plays a crucial role in orchestrating an appropriate and effective antimicrobial immune response against *T. gondii* infection.

## Materials and methods

### Mouse experiments

All procedures and protocols were approved by the Institutional Animal Care and Use Committee at the University of California, Irvine. Both male and female mice, aged 6–10 weeks, were used in the experiments. Wild-type C57BL/6J (JAX stock #00064), CD45.1 (C57BL/6J-Ptprcem6Lutzy/J, JAX stock #033076) and TREM2 KO mice (JAX stock #027197) were purchased from Jackson Labs to establish colonies, and experiments were conducted on purchased mice and mice bred in-house. Genotyping was confirmed by PCR (S8 Fig). Animal husbandry was provided by ULAR. Mice were infected with *T. gondii* by intraperitoneal (i.p.) injection of 200 GFP-expressing type II *Prugniaud* tachyzoites suspended in 200 µL of PBS. Control mice received an i.p. injection of an equivalent volume of sterile PBS (Corning). Mouse weights were tracked, and euthanasia was performed using CO_2_ administration followed by cervical dislocation.

### Cell and parasite culture

*T. gondii* tachyzoites expressing GFP or tdTomato (type II *Prugniaud* strain) were serially passaged in human foreskin fibroblasts (HFFs). To maintain high infectivity, all parasites used for *in vivo* and *in vitro* infection experiments were harvested when the parasites were still intracellular, and they were mechanically lysed out of HFFs before natural egress, as previously described [55]. Bone marrow-derived macrophages (BMDMs) were generated by isolating bone marrow cells from mouse femurs and lysing RBCs using ACK lysis buffer (Thermofisher). The cells were resuspended in macrophage media (MaM), including 5% FBS, 1% Penicillin Streptomycin, and 1% Macrophage Growth Supplement (ScienCell), transferred to non-TC treated Petri dishes (Corning), and differentiated for 7 days, with media replacement after 3 days.

### qPCR

DNA was isolated from liver tissue with the AllPrep DNA/RNA kit (QIAGEN) according to the manufacturer’s protocol. Primers targeting the *T. gondii* B1 gene (forward: CAGATGTGCTAAAGGCGTCA, reverse: GCCCTAGACAGACAGCGAAC) and GAPDH (forward: GCATGGCCTTCCGTGTTC, reverse: CCCAGCTCTCCCCATACATA), a housekeeping gene, were utilized to quantify parasite burden. DNA and primers were incubated with iTaq Universal SYBR Green qPCR Mastermix (Biorad) on a Biorad iCycler. All PCR was performed in triplicate, and transcript abundance was normalized to *gapdh* expression.

### Mouse IVIS procedure

Mice were infected as described, with firefly luciferase-expressing tachyzoites (*Prugniaud*). Every 2–3 days, mice were injected with 15 mg/ml D-luciferin (ThermoFisher) 10 min prior to imaging. Animals were imaged in a dark, light-proof chamber using an IVIS Lumina (Perkin Elmer) CCD camera. Anesthesia with isoflurane via nose cone was maintained throughout the imaging procedure. The camera was controlled using Living Image software. Exposure time was set to 1 sec, 2 min, or 5 min, and regions of interest were drawn in terms of radiance/photons. All data were exported to Microsoft Excel or PRISM (GraphPad) for further analysis.

### Liver histology and pathology scoring

For immunofluorescence imaging, left liver lobes from PBS-injected or *T. gondii-*infected mice were placed in 4% paraformaldehyde overnight, then in 30% sucrose for 3–5 days at 4˚C, protected from light. 12 µm sections were cut on a cryostat, then stained with anti-IBA1 antibodies, DAPI, and tomato lectin. Slides were visualized using a Leica TCS SP8 at the UCI Optical Biology Core Facility, and quantified using ImageJ (Fiji). For histological analysis, left liver lobes were placed in 10% neutral-buffered formalin for 48 hr. Organs were embedded in paraffin, sectioned, and stained with hematoxylin and eosin (H&E) by the Experimental Tissue Resource Core at UC Irvine. The extent of liver pathology was blind scored by a pathologist. Scoring was based on the assessment of liver sections within the sample set and reflect semi-quantitative thresholds distinguishing minimal, moderate, and severe injury: scores of less than 2.5 indicate < 8 foci of inflammatory cells and uninjured hepatocytes, scores from 2.5-6 indicate widespread infiltration of lymphocytes and minor to moderate vacuolization of hepatocytes, and scores above 6 indicate widespread hepatocyte apoptosis and liver infarction. The scoring system represents gradations of injury severity within our dataset rather than universally applicable cutoffs.

### Flow cytometry

Peritoneal exudate was harvested via peritoneal lavage following euthanasia. Blood was taken via cardiac puncture into EDTA anticoagulant tubes (Greiner Bio-one). RBCs were lysed using ACK lysis buffer (Thermofisher). Blood and peritoneal exudate cells were resuspended in staining buffer (1X PBS + 3% FBS + 0.05% EDTA). Cells were surface-stained with directly conjugated antibodies (S9 Fig) and 10% TruStain FcX (Biolegend) to block non-specific binding. Samples were then stained with propidium iodide to determine viability and run on a Novocyte 3000 flow cytometer (Agilent). The data were analyzed via FlowJo software (BD Biosciences).

### Multiplex cytokine ELISA

Mice were infected as described for 3 or 7 days, and peritoneal exudate was harvested. The peritoneal exudate was centrifuged, and the supernatant was subjected to the LEGENDplex Mouse Inflammation Panel (Biolegend). Samples were analyzed on a Novocyte 3000 flow cytometer (Agilent).

### Bone marrow chimera

CD45.1^+^ C57BL/6 and CD45.2^+^ TREM2 KO mice bred in-house were irradiated with 850 cGy using a X-RAD 320 (Precision). Mice were then retro-orbitally injected with 5x10^6^ bone marrow cells isolated from donor mice in a 150 µl volume. Transplanted mice were maintained on antibiotic chow for 3 weeks, then switched to normal chow. Chimerism was confirmed via analysis of granulocytes in peripheral blood from the tail vein before infection experiments were conducted. Chimeric mice were infected i.p. with 200 GFP^+^ parasites, and at 3 dpi, they were euthanized, and the peritoneal exudate, omentum, and liver were collected. Samples were stained as above and run on a Novocyte 3000 flow cytometer.

### Macrophage imaging experiments

BMDMs were seeded on day 6 post-differentiation onto coverslips (Fisher) overnight. The cells were then infected with GFP^+^ or TdT^+^ parasites at MOI = 2 or 4. Coverslips were fixed in 4% paraformaldehyde (Sigma) for 10–15min, then permeabilized/blocked in PBS + 0.3% Triton-X + 3% BSA for 30 min. Cells were stained with rabbit anti-F4/80 (Thermofisher) or rat anti-LAMP1 (Biolegend) antibodies overnight at 4˚ C, then secondary stained with anti-rat Alexa Fluor 594 and anti-rabbit Alexa Fluor 647 (Thermofisher). Slides were visualized using a Leica TCS SP8 at the UCI Optical Biology Core Facility and quantified using ImageJ (Fiji). Parasite burden was determined by counting the number of intact parasites (non-degraded, bright) per 100 cells. F4/80 was used to determine individual macrophage ROIs, and LAMP1 MFI per cell was then quantified. For ERK inhibitor experiments, prior to infection, macrophages were treated for 2 hr with 1 μM ERK 1/2 inhibitor SCH772984 (Selleck Chemicals), then washed thoroughly with PBS before parasite addition.

### *In vitro* phagocytosis assays

BMDMs were seeded overnight in a 24-well plate (Thermofisher), then incubated overnight. *T. gondii* tachyzoites were pre-treated with 5 µM 4-BPB (Sigma) for 15 min or DMSO control, then washed extensively to remove excess drug before incubation with the BMDMs. Following 1 hr or 24 hr, cells were harvested for flow cytometry and stained with Zombie NIR for viability. For imaging experiments, cells on cover slips were fixed using 4% PFA for 15 min and permeabilized with Cytofix (BD Biosciences) for 1 hr. Cells were stained with anti-LAMP1 (Cell Signaling) and anti-F4/80 (Thermofisher). Slides were visualized using a Leica TCS SP8 at the UCI Optical Biology Core Facility, and quantified using ImageJ (Fiji). The phagocytic index was calculated as follows: (# tdTomato^+^ BMDM (4-BPB condition)) / (# tdTomato^+^ BMDM (vehicle condition)) x 100. For pHrodo experiments, BMDMs were seeded into a 96-well plate in triplicate and cultured overnight. The following day, the cells were incubated with pHrodo-tagged *E. coli* particles (Thermofisher) for 5 hr in a BioTek Cytation 5 Plate Reader (Agilent). The pHrodo MFI was determined every 15 min.

### RNA sequencing

3 C57Bl/6 and 4 TREM2 KO mice were infected i.p. with 200 *T. gondii* tachyzoites. At 3 dpi, the peritoneal exudate was harvested, and 1x10^5^ monocytes/mouse were sorted using a FACSAria Fusion sorter (BD Biosciences) at the UC Irvine Institute for Immunology Flow Cytometry Facility. The gating strategy used for sorting is in S7B Fig. Although the recruitment of monocytes to the peritoneal cavity was lower in the TREM2 KO mice, we sorted and extracted RNA from the same number of CD45^+^CD11b^+^Ly6C^+^ cells from both genotypes of infected mice to ensure comparable coverage during RNA-sequencing. Total RNA was isolated from the monocytes using the RNeasy mini kit (QIAGEN), and libraries were prepared using the NEBNext Ultra II RNA Library Prep Kit for Illumina (New England Biolabs). Libraries were sequenced at the UC Irvine Genomics Research and Technology Hub. Quality control and adaptor trimming was performed using Trimmomatic. Surviving reads were aligned to the mouse GRCm39 primary assembly (Gencode release 27) using STAR aligner (Version 2.5.4b). Reads were counted using FeatureCounts, a part of the Subread package [56]. Further analyses were performed in MATLAB, and these included differential gene expression analyses and data visualization. All of the scripts used for data analysis have been deposited in Github (https://github.com/hdebray527/trem2tgondii/blob/main/README.md). Genes that were differentially expressed between clusters were identified by a negative binomial exact test, followed by Benjamini-Hochberg false-detection rate (FDR) correction. A gene was considered differentially expressed if the FDR-corrected p-value was below 0.05, and the absolute fold change was more than 1.5. Functional annotation of DEGs was performed using g:Profiler (https://biit.cs.ut.ee/gprofiler/gost), which provides enrichment analysis across multiple functional databases. Differentially expressed genes were queried against the Gene Ontology (biological process, molecular function, and cellular component), KEGG, and Reactome pathway databases. Enrichment significance was assessed using the g:SCS multiple testing correction method, with adjusted p-value (FDR) < 0.05 considered significant. Further analyses and visualization of the data were performed using Matlab r2024b.

### Statistical analysis

Student’s t test and two-way ANOVA analyses with Tukey post-hoc tests were performed in GraphPad Prism. P < 0.05 was defined as the threshold for statistically significant differences. For the RNA sequencing data, the adjusted p-value according to size factor estimation and rlog transformation and a threshold log2 fold change greater than 1.5 or lower than -1.5 were used to determine significantly different genes.

## Supporting information

S1 Fig Cytokine concentrations in WT and TREM2 KO mice. C57BL/6 WT and TREM2 KO mice were injected i.p. with PBS (A) or 200 type II *T. gondii* tachyzoites (B). Peritoneal exudate was harvested after 3 days and analyzed by the LegendPlex multiplex cytokine assay for 13 analytes, with higher concentration analytes graphed on the left and lower concentrations analytes on the right. LOD: limit of detection. n = 6 mice per genotype per condition.
(PDF)

S2 Fig Peritoneal exudate cells at 3 days post-infection. A) C57BL/6 WT mice were injected with PBS or infected with 200 type II GFP-expressing *T. gondii* and the peritoneal exudate cells (PECs) were harvested and analyzed at 3 dpi. Gating strategy for PECs after staining with propidium iodide, anti-CD45, anti-CD11b, anti-CD3, anti-CD19, anti-F4/80, anti-Ly6G, and anti-Ly6C. B) Numbers of GFP^+^ (infected) cells in each cell population in the PECs. n = 12 mice per group, by Student’s t-test. ns: not significant.
(PDF)

S3 Fig Cells in the omentum at 3 dpi. A) Gating strategy for omentum cells that were stained with propidium iodide, anti-CD45, anti-CD11b, anti-CD3, anti-CD19, anti-F4/80, anti-Ly6G, and anti-Ly6C. B) Numbers of GFP^+^ (infected) cells in each cell population in the omentum. n = 5–8 mice per group, *p < 0.05 by Student’s t-test.
(PDF)

S4 Fig Engraftment of donor bone marrow cells in recipient mice from bone marrow chimera experiments. A) Representative flow plots of gating scheme for identifying Gr-1^+^ granulocytes in blood. B) Quantification of chimerism of Gr-1^+^ cells in the blood of control and chimeric mice. WT cells express CD45.1, whereas TREM2 KO cells express CD45.2. C) Representative flow plots of large peritoneal macrophages in WT◊TREM2 KO and TREM2 KO◊WT groups. D) Reconstitution of large peritoneal macrophages in WT and TREM2 KO mice (un-reconstituted and not irradiated), WT◊WT and TREM2 KO◊TREM2 KO (reconstituted with donor bone marrow from the same genotype), and WT◊TREM2 KO or TREM2 KO◊WT (reconstituted with donor bone marrow from the opposite genotype). E) Percent of GFP^+^ (infected) cells in the PECs and omentum of WT and TREM2 KO mice reconstituted with donor bone marrow from the same genotype. n = 2–6 mice/group, *p < 0.05, ****p < 0.0001 by one-way ANOVA.
(PDF)

S5 Fig Phagocytosis of 4-BPB-treated (invasion-deficient) *T. gondii.* A) Representative flow plots of WT BMDMs incubated with either vehicle- or 4-BPB-treated *T. gondii* at 1 hpi and 24 hpi. B) Percent of tdTomato^+^ cells following 1 hr or 24 hr incubation with vehicle- or 4-BPB treated parasites. C) HFF plaque assay with parasites treated with vehicle control (left) or 4-BPB (right) and harvested from macrophages. D) WT or TREM2 KO BMDMs were cultured with pHrodo *E. coli* bioparticles in a plate reader. Fluorescence was measured every 15 min for 5 hr. ****p > 0.0001.
(PDF)

S6 Fig LAMP1 expression following ERK inhibition in WT and TREM2 KO macrophages. A) Representative images of WT or TREM2 KO BMDMs incubated with ERK inhibitor and parasites at MOI = 2 for 1 hpi. Images taken at 63X, 2–4 FOV/group. B) LAMP1 MFI/cell was determined using F4/80 to define the cell borders. ****p < 0.0001, by one-way ANOVA. C) Parasite numbers per 100 cells were quantified, n = 5–6 experiments, **p < 0.01, *p < 0.05 by one-way ANOVA.
(PDF)

S7 Fig RNAseq experimental pipeline and analysis. C57BL/6 WT or TREM2 KO mice were injected with PBS or infected with 200 type II *T.* gondii. At 3 dpi, peritoneal exudate cells were harvested, sorted, and analyzed by RNAseq. A) Schematic of RNAseq experimental workflow and data analysis, created in BioRender. B) Representative gating scheme for cell sorting. PECs were stained with antibodies against CD11b, Ly6C, Ly6G, and propidium iodide as a viability stain. 1x10^5^ CD11b^+^Ly6C^+^Ly6G^-^ monocytes were sorted on a FACS Aria Fusion cell sorter, then immediately processed for RNA isolation, library prep, and downstream analysis. The average time to sort 10^5^ monocytes was 2–3:30 min from WT mice and 4–8 min from TREM2 KO mice. C) 3D Principal Component Analysis (PCA) of RNAseq experiment. Purple dots represent WT animals, and yellow dots represent TREM2 KO animals.
(PDF)

S8 Fig Confirmation of TREM2 deficiency in TREM2 KO mice. A) Genomic DNA sequence of *Trem2* near exon 2. Primer sequences were provided by M. Sasner for TREM2 KO mice (JAX #027197). B) Genomic DNA was isolated from ear punches from TREM2 KO and WT mice. Genotyping PCR was conducted and shows a 254 bp product in four TREM2 KO mice and a 431 bp product in two WT mice. NTC = no template control.
(PDF)

S9 Fig Antibodies used in flow cytometry experiments. (PDF)

S1 File Raw Gel file of the gel in S8 Fig. (PDF)
