# Supplementary material for: TREM2 plays a critical role in innate immune defense against acute Toxoplasma gondii infection by promoting macrophage antimicrobial activities
Source: PLoS Pathog. 2026 Aug 5;22(8):e1014462. doi: 10.1371/journal.ppat.1014462 (PMC13440800; doi:10.1371/journal.ppat.1014462)
Supplement: Supplementary file 1 [file ppat.1014462.s001.pdf]

S1 Figure

A

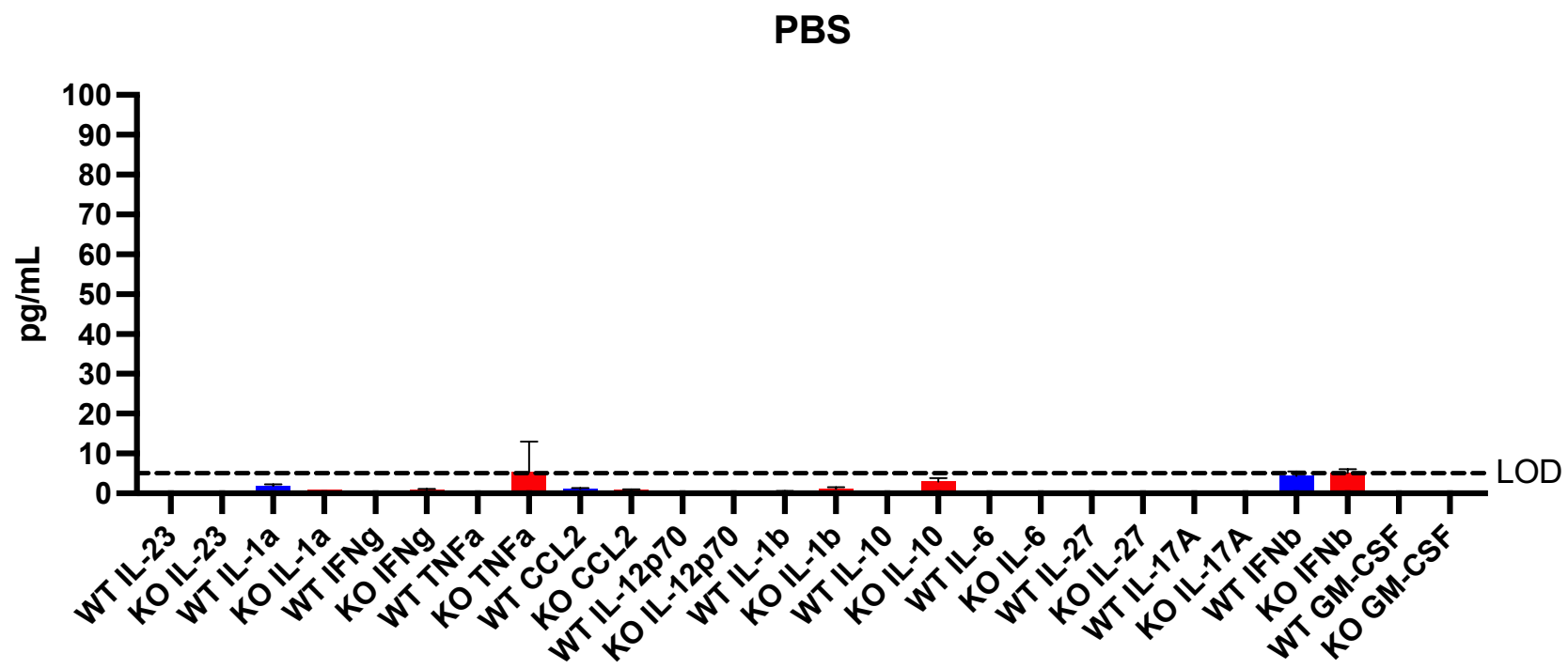

B

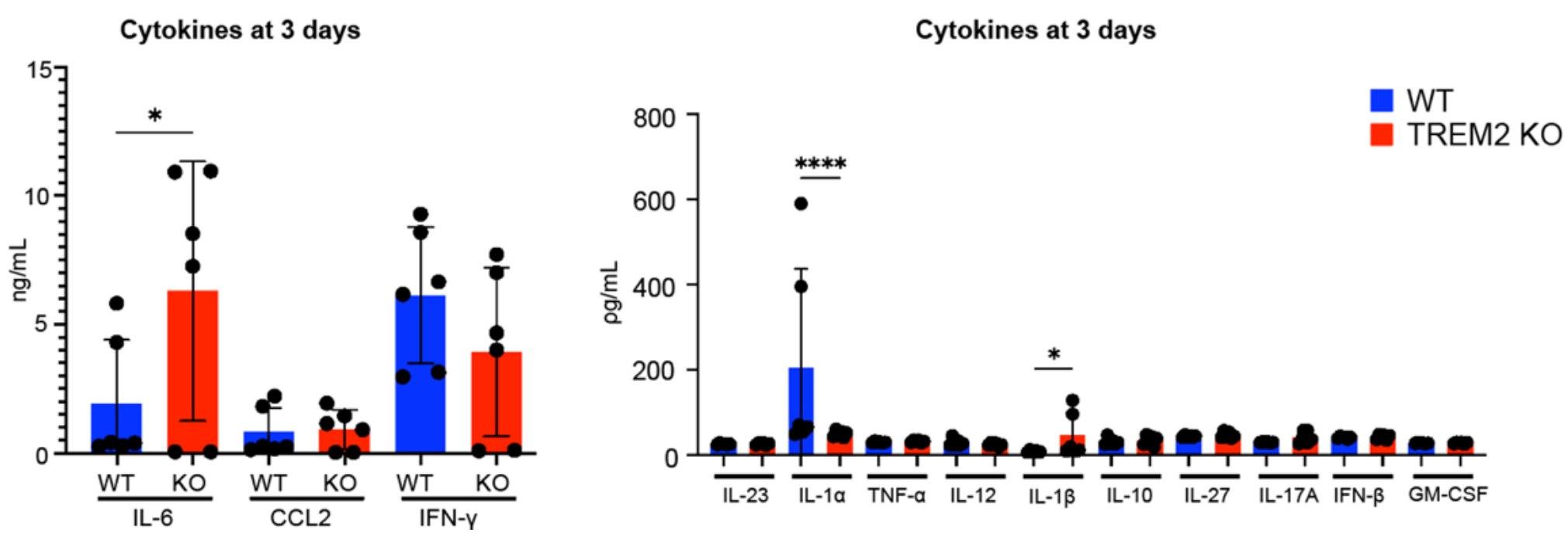

S1 Fig. Cytokine concentrations in WT and TREM2 KO mice. C57BL/6 WT and TREM2 KO mice were injected i.p. with PBS (**A**) or 200 type II *T. gondii* tachyzoites (**B**). Peritoneal exudate was harvested after 3 days and analyzed by the LegendPlex multiplex cytokine assay for 13 analytes, with higher concentration analytes graphed on the left and lower concentrations analytes on the right. LOD: limit of detection. n=6 mice per genotype per condition.
