# Supplementary material for: TREM2 plays a critical role in innate immune defense against acute Toxoplasma gondii infection by promoting macrophage antimicrobial activities
Source: PLoS Pathog. 2026 Aug 5;22(8):e1014462. doi: 10.1371/journal.ppat.1014462 (PMC13440800; doi:10.1371/journal.ppat.1014462)
Supplement: Supplementary file 2 [file ppat.1014462.s002.pdf]

S2 Figure

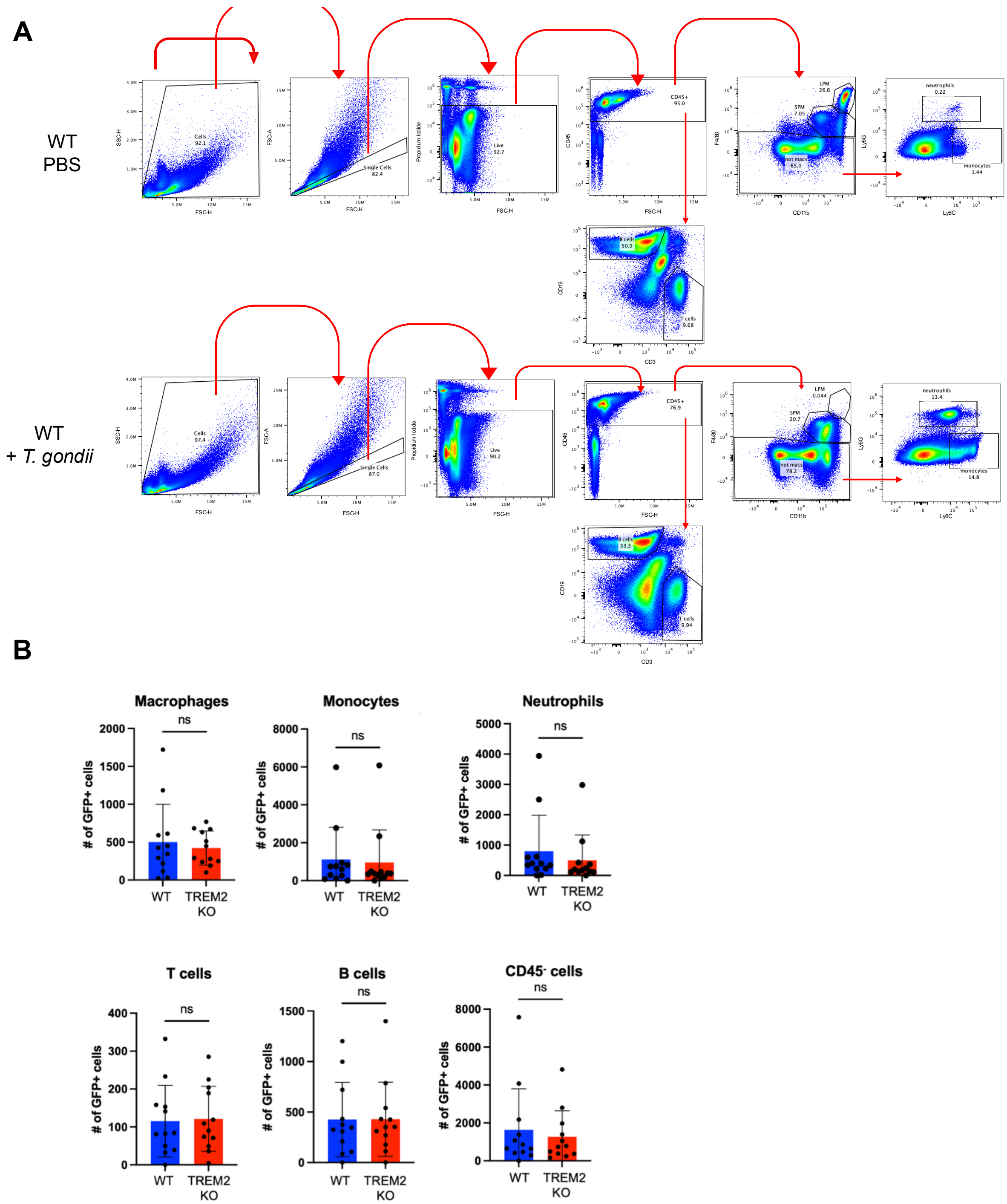

S2 Fig. Peritoneal exudate cells at 3 days post-infection. A) C57BL/6 WT mice were injected with PBS or infected with 200 type II GFP-expressing *T. gondii* and the peritoneal exudate cells (PECs) were harvested and analyzed at 3 dpi. Gating strategy for PECs after staining with propidium iodide, anti-CD45, anti-CD11b, anti-CD3, anti-CD19, anti-F4/80, anti-Ly6G, and anti-Ly6C. **B)** Numbers of GFP^+ (infected) cells in each cell population in the PECs. n = 12 mice per group, by Student's t-test. ns: not significant.
