# Supplementary material for: TREM2 plays a critical role in innate immune defense against acute Toxoplasma gondii infection by promoting macrophage antimicrobial activities
Source: PLoS Pathog. 2026 Aug 5;22(8):e1014462. doi: 10.1371/journal.ppat.1014462 (PMC13440800; doi:10.1371/journal.ppat.1014462)
Supplement: Supplementary file 3 [file ppat.1014462.s003.pdf]

S3 Figure

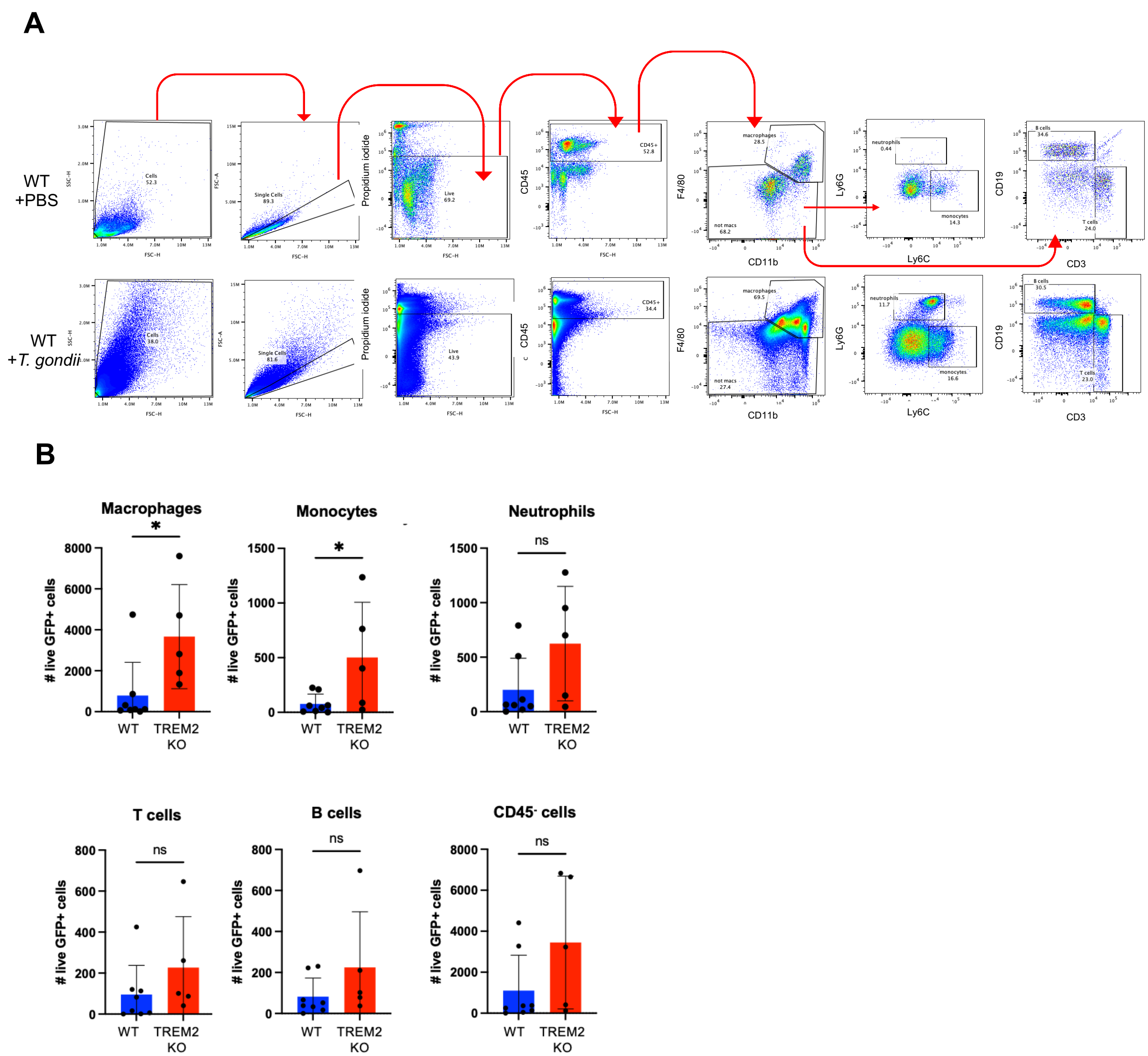

S3 Fig. Cells in the omentum at 3 dpi. A) Gating strategy for omentum cells that were stained with propidium iodide, anti-CD45, anti-CD11b, anti-CD3, anti-CD19, anti-F4/80, anti-Ly6G, and anti-Ly6C. **B)** Numbers of GFP^{+} (infected) cells in each cell population in the omentum. n = 5-8 mice per group, *p<0.05 by Student's t-test.
