# Supplementary material for: TREM2 plays a critical role in innate immune defense against acute Toxoplasma gondii infection by promoting macrophage antimicrobial activities
Source: PLoS Pathog. 2026 Aug 5;22(8):e1014462. doi: 10.1371/journal.ppat.1014462 (PMC13440800; doi:10.1371/journal.ppat.1014462)
Supplement: Supplementary file 4 [file ppat.1014462.s004.pdf]

S4 Figure

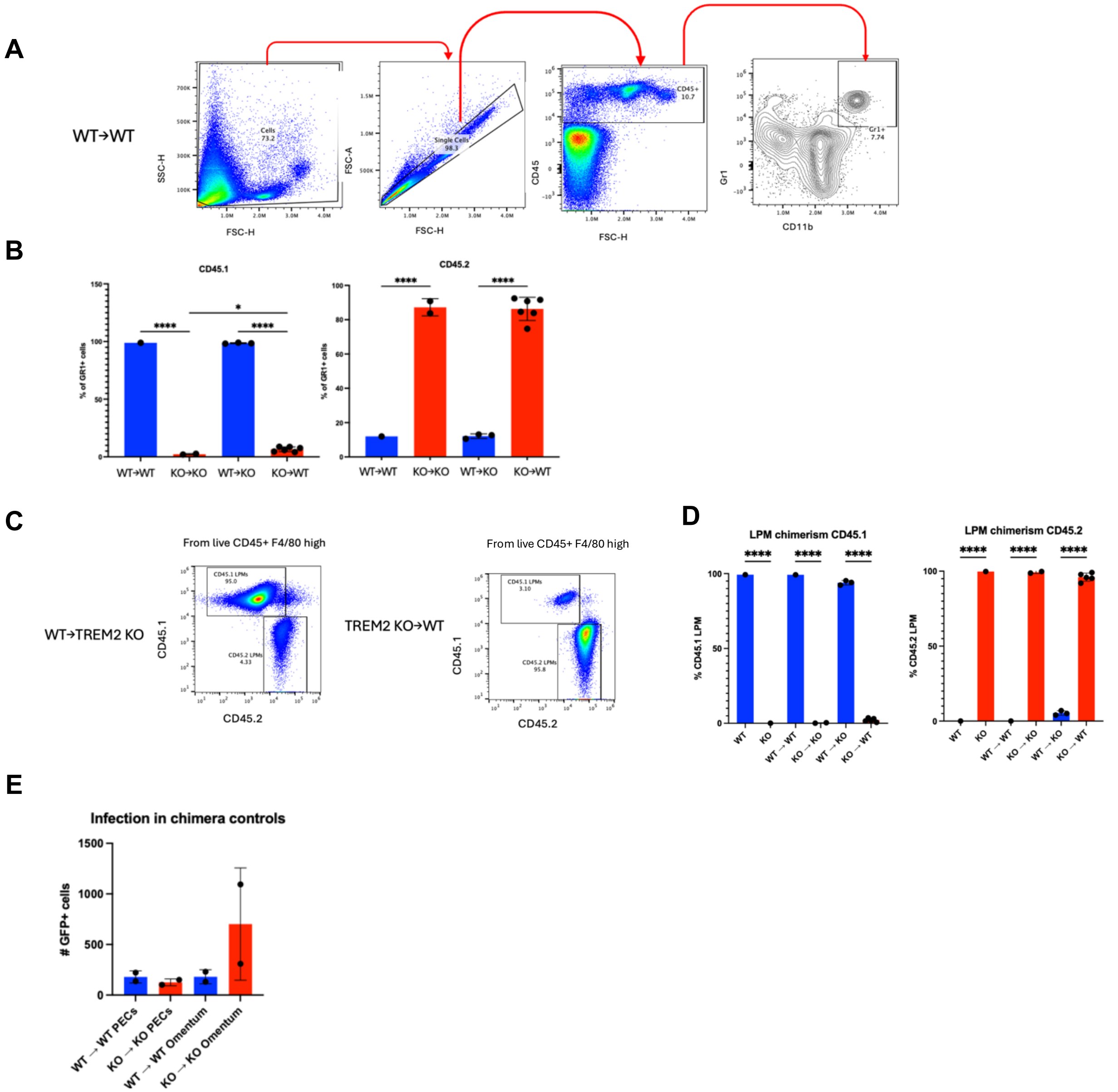

S4 Fig. Engraftment of donor bone marrow cells in recipient mice from bone marrow chimera experiments. A) Representative flow plots of gating scheme for identifying $Gr\text{-}1^+$ granulocytes in blood. **B)** Quantification of chimerism of $Gr\text{-}1^+$ cells in the blood of control and chimeric mice. WT cells express CD45.1, whereas TREM2 KO cells express CD45.2. **C)** Representative flow plots of large peritoneal macrophages in WT→TREM2 KO and TREM2 KO→WT groups. **D)** Reconstitution of large peritoneal macrophages in WT and TREM2 KO mice (un-reconstituted and not irradiated), WT→WT and TREM2 KO→TREM2 KO (reconstituted with donor bone marrow from the same genotype), and WT→TREM2 KO or TREM2 KO→WT (reconstituted with donor bone marrow from the opposite genotype). **E)** Percent of GFP^+ (infected) cells in the PECs and omentum of WT and TREM2 KO mice reconstituted with donor bone marrow from the same genotype. n=2-6 mice/group, *p<0.05, ****p<0.0001 by one-way ANOVA.
