# Supplementary material for: TREM2 plays a critical role in innate immune defense against acute Toxoplasma gondii infection by promoting macrophage antimicrobial activities
Source: PLoS Pathog. 2026 Aug 5;22(8):e1014462. doi: 10.1371/journal.ppat.1014462 (PMC13440800; doi:10.1371/journal.ppat.1014462)
Supplement: Supplementary file 5 [file ppat.1014462.s005.pdf]

S5 Figure

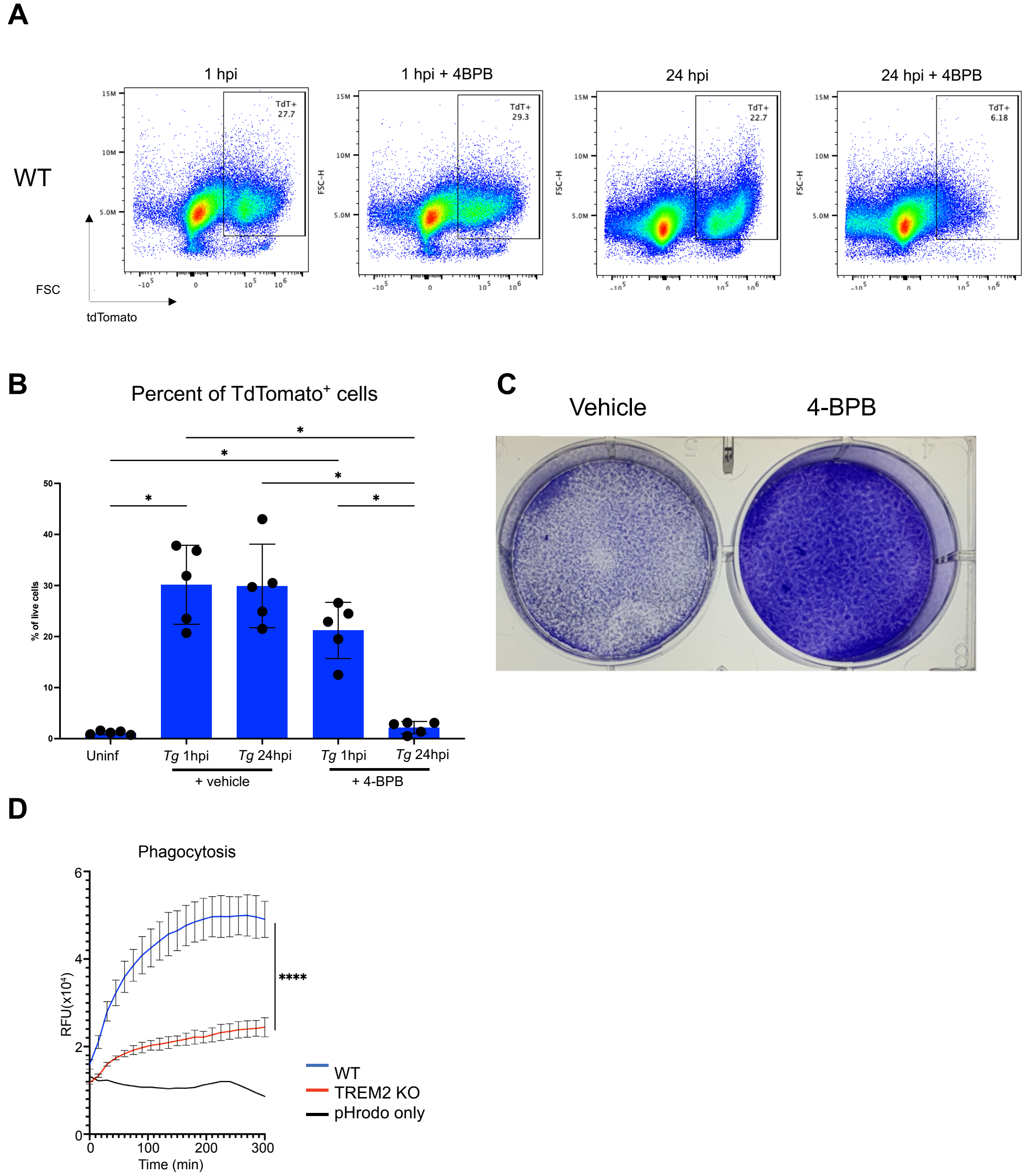

S5 Fig. Phagocytosis of 4-BPB-treated (invasion-deficient) *T. gondii*. A) Representative flow plots of WT BMDMs incubated with either vehicle- or 4-BPB-treated *T. gondii* at 1 hpi and 24 hpi. **B)** Percent of tdTomato^{+} cells following 1 hr or 24 hr incubation with vehicle- or 4-BPB treated parasites. **C)** HFF plaque assay with parasites treated with vehicle control (left) or 4-BPB (right) and harvested from macrophages. **D)** WT or TREM2 KO BMDMs were cultured with pHrodo *E. coli* bioparticles in a plate reader. Fluorescence was measured every 15 min for 5 hr. ****$p > 0.0001$.
