# Supplementary material for: TREM2 plays a critical role in innate immune defense against acute Toxoplasma gondii infection by promoting macrophage antimicrobial activities
Source: PLoS Pathog. 2026 Aug 5;22(8):e1014462. doi: 10.1371/journal.ppat.1014462 (PMC13440800; doi:10.1371/journal.ppat.1014462)
Supplement: Supplementary file 6 [file ppat.1014462.s006.pdf]

S6 Figure

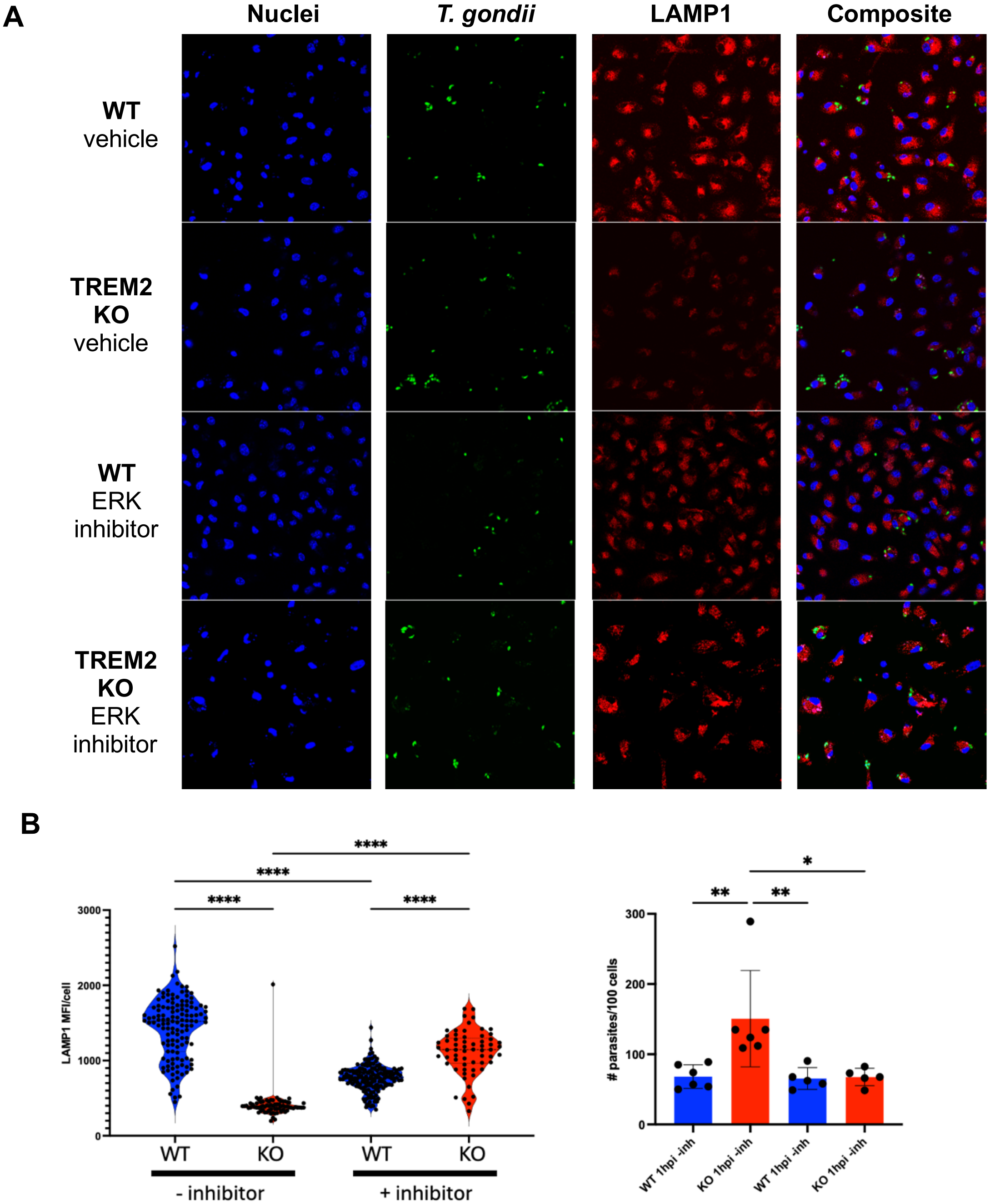

S6 Fig. LAMP1 expression following ERK inhibition in WT and TREM2 KO macrophages. A) Representative images of WT or TREM2 KO BMDMs incubated with ERK inhibitor and parasites at MOI = 2 for 1 hpi. Images taken at 63X, 2-4 FOV/group. **B)** LAMP1 MFI/cell was determined using F4/80 to define the cell borders. ****p<0.0001, by one-way ANOVA. **C)** Parasite numbers per 100 cells were quantified, n=5-6 experiments, **p<0.01, *p<0.05 by one-way ANOVA.
