# Supplementary material for: TREM2 plays a critical role in innate immune defense against acute Toxoplasma gondii infection by promoting macrophage antimicrobial activities
Source: PLoS Pathog. 2026 Aug 5;22(8):e1014462. doi: 10.1371/journal.ppat.1014462 (PMC13440800; doi:10.1371/journal.ppat.1014462)
Supplement: Supplementary file 7 [file ppat.1014462.s007.pdf]

S7 Figure

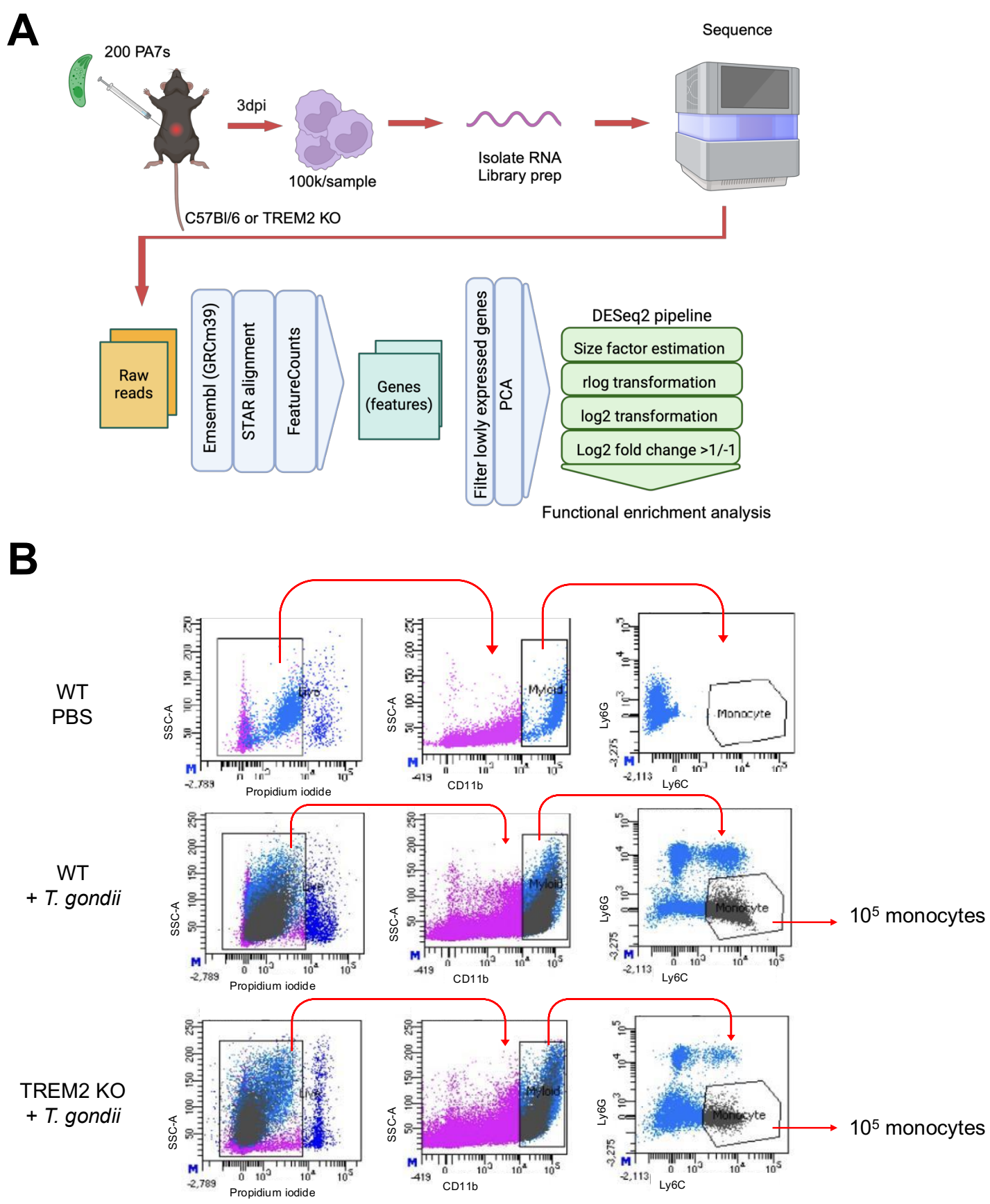

S7 Fig. RNAseq experimental pipeline and analysis. C57BL/6 WT or TREM2 KO mice were injected with PBS or infected with 200 type II *T.* gondii. At 3 dpi, peritoneal exudate cells were harvested, sorted, and analyzed by RNAseq. **A)** Schematic of RNAseq experimental workflow and data analysis. Created in BioRender. Lab, L. (2025) https://BioRender.com/3t6m61f. **B)** Representative gating scheme for cell sorting. PECs were stained with antibodies against CD11b, Ly6C, Ly6G, and propidium iodide as a viability stain. 1x10^5 CD11b$^+$Ly6C$^+$Ly6G$^-$ monocytes were sorted on a FACS Aria Fusion cell sorter, then immediately processed for RNA isolation, library prep, and downstream analysis. The average time to sort 10^5 monocytes was 2-3:30 min from WT mice and 4-8 min from TREM2 KO mice. **C)** 3D Principal Component Analysis (PCA) of RNAseq experiment. Purple dots represent WT animals, and yellow dots represent TREM2 KO animals.
