# Supplementary material for: TREM2 plays a critical role in innate immune defense against acute Toxoplasma gondii infection by promoting macrophage antimicrobial activities
Source: PLoS Pathog. 2026 Aug 5;22(8):e1014462. doi: 10.1371/journal.ppat.1014462 (PMC13440800; doi:10.1371/journal.ppat.1014462)
Supplement: Supplementary file 8 [file ppat.1014462.s008.pdf]

S8 Figure

A

TREM2 Exon 2

Forward primer

ATGACCATGCACACGCTATGCTCCCTGCACTCCTGGAACTGCTTCCAAGCAAGTGG
CTGTCTCCTCTGCAGCCCTGTC(CCAAGCCCTCAACACCACGGTGCTGCAGGGCAT
GGCCGGCCAGTCCTTGAGGGTGTCATGTACTTATGACGCCTTGAAGCACTGGGGG
AGACGCAAGGCCTGGTGTCGGCAGCTGGGTGAGGAGGGCCCATGCCAGCGTGTG
GTGAGCACACACGGTGTGTGGCTGCTGGCCTTC)CTGAAGAAGCGGAATGGGAGC
ACAGTCATCGCAGATGACACCCTTGCTGGAACCGTCACCATCACTCTGAAGAACCT
CCAAGCCGGTGACGCGGGCCTCTACCAGTGTCAGAGTCTCCGAGGCCGAGAGGC
TGAGGTCCTGCAGAAAGTACTGGTGGAGGTGCTGGAGGGTGAGTA

Reverse primer

Sequence in red is deleted in the TREM2 KO
WT product = 431 bp
KO product = 254 bp

B

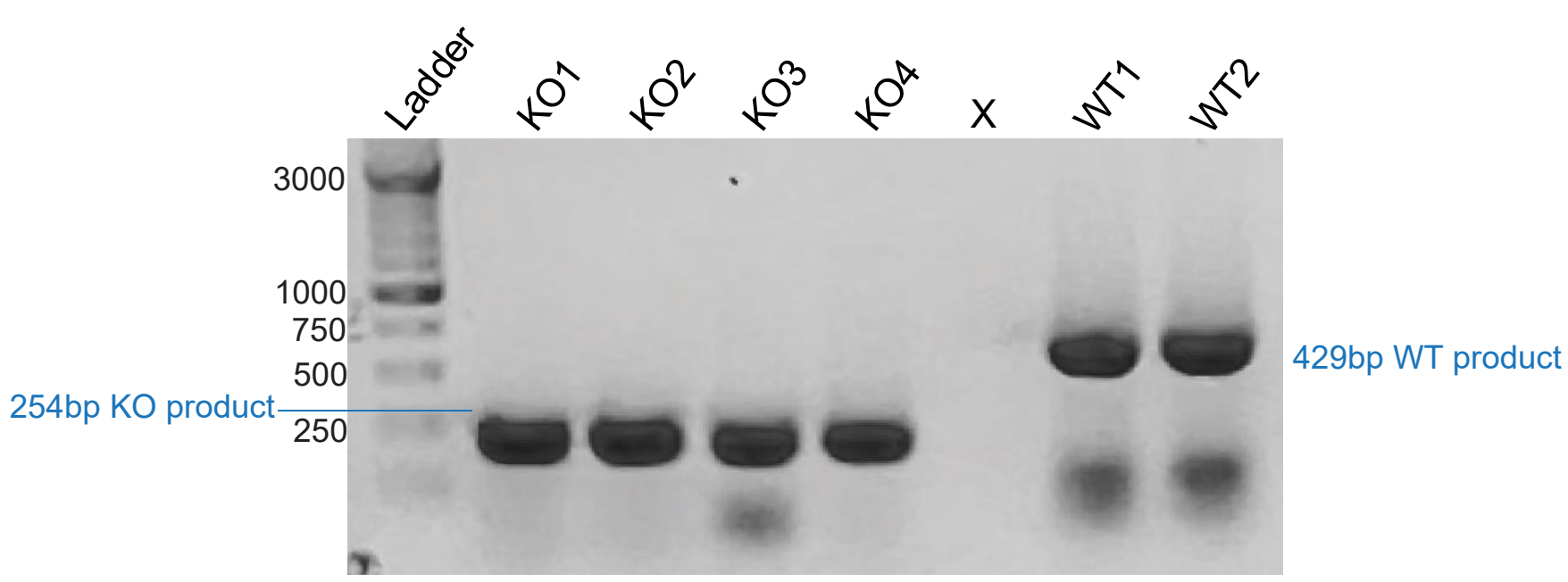

S8 Fig. Confirmation of TREM2 deficiency in TREM2 KO mice. A) Genomic DNA sequence of *Trem2* near exon 2. Primer sequences were provided by M. Sasner for TREM2 KO mice (JAX #027197). **B)** Genomic DNA was isolated from ear punches from TREM2 KO and WT mice. Genotyping PCR was conducted and shows a 254 bp product in four TREM2 KO mice and a 431 bp product in two WT mice. X = empty well.
