# Supplementary material for: TREM2 plays a critical role in innate immune defense against acute Toxoplasma gondii infection by promoting macrophage antimicrobial activities
Source: PLoS Pathog. 2026 Aug 5;22(8):e1014462. doi: 10.1371/journal.ppat.1014462 (PMC13440800; doi:10.1371/journal.ppat.1014462)
Supplement: Supplementary file 9 [file ppat.1014462.s009.pdf]

S9 Figure

Antibody Panel	Source	Identifier
Brilliant Violet 785 anti-mouse CD45	Biolegend	103149
Brilliant Violet 605 anti-mouse CD11b	Biolegend	101257
APC anti-mouse F4/80	Biolegend	123116
PerCP/Cyanine 5.5 anti-mouse Ly6C	Biolegend	128012
Brilliant Violet 510 anti-mouse Ly6G	Biolegend	127633
PE/Cy7 anti-mouse CD19	Biolegend	115520
APC-Cy7 anti-mouse CD3	Biolegend	100222
TruStain FcX (anti-mouse CD16/32)	Biolegend	101320
Propidium Iodide	eBiosciences	p3566

S9 Fig. Antibodies used in flow cytometry experiments.
